# Lack of SIRP‐alpha reduces lung cancer growth in mice by promoting anti‐tumour ability of macrophages and neutrophils

**DOI:** 10.1111/cpr.13361

**Published:** 2022-11-23

**Authors:** Linyue Pan, Bin Wang, Mengjie Chen, Yuan Ma, Bo Cui, Zhihong Chen, Yuanlin Song, Lijuan Hu, Zhilong Jiang

**Affiliations:** ^1^ Department of Pulmonary Medicine, Zhongshan Hospital Fudan University Shanghai China; ^2^ Department of Thoracic Surgery, Huadong Hospital Fudan University Shanghai China; ^3^ Shanghai Key Laboratory of Lung Inflammation and Injury Shanghai China

## Abstract

**Objectives:**

Signal regulatory protein‐alpha (SIRPα) is a transmembrane glycoprotein specifically expressed on myeloid cells. Blockade of SIRPα/CD47 interaction is effective in combinational therapy of some cancers. This study aimed to explore into the role and underlying molecular mechanisms of SIRPα in lung cancer growth.

**Materials and Methods:**

A mouse model with lung cancer in wild‐type (WT) and SIRPα‐knockout mouse (KO) mice was established by subcutaneous injection of Lewis murine lung cancer cells (LLC). Circulating monocytes and neutrophils were depleted in mice by intraperitoneal administration of clodronate liposomes and anti‐Ly6G antibody, respectively. Phenotypes and phagocytosis of macrophages and neutrophils were analysed by flow cytometry. Transwell assay was used to analyse LLC cells migration and invasion.

**Results:**

Lack of SIRPα inhibited LLC cells growth in KO mice, associated with reduced infiltrating PD‐1^+^CD8^+^ T cells and production of IL‐6 from infiltrating macrophages and neutrophils in tumour tissues. Depletion of circulating monocytes and neutrophils reduced LLC cells growth in WT mice, which was abolished in KO mice. Studies in vitro showed that lack of SIRPα increased M1/M2 ratio, and reduced LLC cell migration and invasion via attenuated IL‐6 secretion. Lack of SIRPα expression in neutrophils effectively increased the cytotoxic activity to LLC cells in vitro.

**Conclusions:**

Lack of SIRPα suppressed lung cancer cell growth in mice, dependent on circulating macrophages and neutrophils, in association with improved phagocytosis and reduced IL‐6 expression.

## Graphical abstract

Targeting SIRPα alone was qualified to inhibit LLC growth. SIRPα inhibits macrophages and neutrophils phagocytosis. Tumour‐derived mediators induce M2 cell polarisation, IL‐6 expression in macrophages and neutrophils through SIRPα/SHP‐1/p38 MAPK/STAT3 signalling. IL‐6 induces epithelial–mesenchymal transition of LLC cells and PD‐1 expression in CD8^+^ T cells.

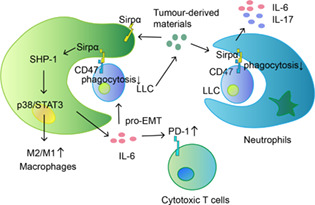

## 1 INTRODUCTION

Lung cancer ranks first in mortality and second in the prevalence of malignant cancer worldwide in 2021.^1^ To date, targeting immune checkpoints such as programmed death‐1 (PD‐1), and cytolytic T lymphocyte‐associated antigen‐4 (CTLA‐4), have become effective therapeutic approaches among some lung cancer patients.^2^, ^3^ However, cohorts of patients have developed immune resistance to the immune checkpoint inhibitors (ICIs), due to infiltration of tumour‐associated macrophages and neutrophils (TAMs and TANs), immunosuppressive myeloid cells and induction of T cell exclusion.^4^, ^5^, ^6^ In addition, IL‐6 is up‐regulated in most of tumour patients and plays an important role in the modulation of these immunosuppressive cells.^7^, ^8^ It was previously reported that the combined blockade of IL‐6 and CTLA‐4 improved the survival of tumour‐bearing mice by reducing infiltration of PD‐1^+^CD8^+^ T cells and M2 macrophages in the tumour microenvironment (TME).^4^ Thus, IL‐6 has become a useful biomarker in the evaluation of cancer prognosis and effectiveness of anti‐cancer treatment.^9^, ^10^

Myeloid cells are a major component of tumour‐infiltrating cells in TME, contributing to resistance during immunotherapy by increasing the expression of signal regulatory protein‐alpha (SIRPα) and suppressing antitumoral abilities of T and natural killer (NK) cells in TME.^6^, ^8^ SIRPα is a transmembrane glycoprotein specifically expressed on myeloid cells and provides a “do not eat me” signal after engaged with integrin‐associated protein CD47 on tumour cells.^11^, ^12^ CD47 is expressed at a low level in normal cells and upregulated in most of cancer cells.^13^ It was documented that CD47 blockade could trigger macrophage‐mediated elimination of non‐small cell lung cancer (NSCLC) cells with resistant to EGFR tyrosine kinase inhibitors (TKIs), and eliciting synergistic anti‐tumour effect with TKIs.^5^, ^14^ Similar anti‐tumour effects were also studied in an animal tumour model with melanoma, in which synergistic effects were observed in a combinational blockade of CD47 and CTLA‐4.^15^ However, the effects of targeting SIRPα on cancer immunotherapy have not been well investigated so far. Ring NG et al found that bispecific anti‐CD70/SIRPα antibody had additive effects on anti‐human Burkitt's lymphoma xenograft by enhancing antibody‐dependent cellular phagocytosis (ADCP).^16^ Recently, a bidirectional antibody RRx‐001 was developed to block interaction between CD47 and SIRPα, has shown promising therapeutic effects in phase II clinical trials. It also exerted effective anti‐tumour activity in mouse models of lung cancer by increasing polarisation of the high phagocytic M1 phenotype of macrophages (MPs) with minimal toxicity.^17^

It was documented that CD47 was upregulated in patients carrying EGFR mutations and played an important role in immune evasion.^14^ CD47 upregulation in refractory lung tumour models was mediated by TNF‐α/nuclear factor‐κ‐gene binding (NF‐κB1) signal pathway.^5^ However, the role of SIRPα signalling in the progression of lung cancer is not well investigated so far. After engagement with CD47 on tumour cells, SIRPα is activated through phosphorylation of immunoreceptor tyrosine‐based inhibition motifs (ITIM) on its cytoplasmic tail, then recruits and activates downstream Src homology 2 domain‐containing protein tyrosine phosphatase‐1 (SHP‐1) and SHP‐2, subsequently exerts suppressive effects on phagocytosis and activation of MPs.^18^, ^19^ It was previously reported that SIRPα participated in the development of acute lung injury (ALI) by suppressing macrophage phagocytosis. SIRPα‐driven downstream SHP‐1 activation. Suppression of STAT3 and STAT6 signalling participated in suppressing macrophage phagocytosis after SIRPα activation; whereas lack of SIRPα attenuated ALI through improving macrophage phagocytosis.^20^

p38 MAPK and STAT3 signalling are activated in most of cancer cells and participate in cancer cell proliferation and metastases.^21^, ^22^ Suppression of p38 MAPK and STAT3 activation was associated with reduced lung cancer growth in vitro and in animal models.^23^, ^24^ However, the role and downstream signalling pathways of SIRPα in the progression of lung cancer are not well studied so far. We in this study, for the first time, investigated the role of SIRPα in SIRPα knock‐out (KO) mice and found that lack of SIRPα significantly reduced lung cancer cell growth in mice, in association with improved MPs and neutrophils (NPs) phagocytosis and reduced IL‐6 expression in tumour tissues. The beneficial effects were depended on the presence of MPs and NPs in vivo and mediated via SHP‐1/p38 MAPK/STAT3 signalling. Thereby, we confirm that SIRPα is a useful target in the treatment of lung cancer.

## 2 MATERIALS AND METHODS

### 2.1 Mouse tumour model and treatment

SIRPα knock‐out (KO) mice were created by Cyagen biotech company in Suzhou, China as described previously.^20^ SIRPα^−/−^ and SIRPα^−/+^ mice were used as KO mice, while SIRPα^+/+^ mice as WT mice. To establish a mouse tumour model, the left flank of 9–12 weeks old WT and KO C57BL/6 mice were subcutaneously injected with 10^6^ murine Lewis lung carcinoma cell lines (LLC) in 100 μl volume, after the cells were confirmed mycoplasma contamination negative. Upon development of visible tumour, the tumour size was measured every 3 days. Tumour size (mm^3^ = *a* * *b* * *b*/2) and weight (g) were recorded. Tumours were harvested with a diameter <2000 mm. To establish a mouse tumour model depleted of MPs and NPs, 0.75 mg clodronate liposomes/20 g mouse and 0.2 mg anti‐Ly6G (αLy6G) antibody/20 g mouse were respectively intraperitoneally (i.p.) injected into mouse 1 day before tumour inoculation of LLC cells. The reagents were injected every 5 days until the mice were sacrificed. Depletion of MPs and NPs in mice was verified by flow cytometry (FCM) analysis on day 0 and at the end of experiments.

### 2.2 Cell culture and treatment

LLC cells and mouse fibroblast cell lines, L929 were maintained in DMEM and RPMI‐1640 respectively (Hyclon, Logan, Utah), supplemented with 10% foetal bovine serum (FBS, Gibco, Carlsbad, California). The conditional media of LLC (LLC‐CM) and L929 (L929‐CM) were collected at 90% of cell confluence. Bone marrow‐derived macrophages (BMDMs) were collected from mouse femur and tibia, then differentiated under RPMI‐1640 medium supplemented with 30% L929‐CM for 7 days. Tumour‐associated macrophages (TAMs) were obtained by incubation of BMDMs with 50% LLC‐CM for 24 h (Complete RPMI‐1640 media was used as NC‐CM control). To study signalling transduction pathways, WT and KO mice‐derived BMDMs were respectively treated with 5 μM TPI‐1(SHP‐1 inhibitor, MedChemExpress, Monmouth Junction, New Jersey), 5 μM SB203580 (p38 MAPK inhibitor, Absin, Shanghai, China) and 5 μM C188‐9 (STAT3 inhibitor, Calbiochem, St. Louis, Missouri) for 2 h. Cell supernatants and cells were collected for further analysis.

NPs were obtained from bone marrow cells and purified by Percoll gradient (GE Healthcare, Chicago, Illinois). To obtain splenocytes, mouse spleens were collected and followed by digestion with 1 mg/mL collagenase A (Sigma, St. Louis, Missouri) and red cell lysis. The cells were finally maintained in RPMI1640 medium supplied with 10% FBS. To obtain activated T cells, splenocytes were stimulated with phorbol 12‐myristate 13‐acetate (PMA, BioLegend, San Diego, California) for 24 h.

### 2.3 Phagocytosis assay

Phagocytosis of BMDMs or NPs was measured by incubation of BMDMs or NPs with PKH26‐labelled LLC cells (1:2 ratio) for 24 h or with florescence‐labelled beads for 6 h. Phagocytosis index was presented as the percentage of CD11b^+^PKH26^+^ cells/CD11b^+^ cells or Ly6G^+^PKH26^+^ cells/Ly6G^+^ cells after flow cytometry analysis.

### 2.4 Flow cytometry

Exactly 0.5 to 1 × 10^6^ tumour single‐cell suspension or the treated cells were incubated with an antibody cocktail. Following reagents and antibodies were used for flow cytometry analysis, including Zombie Red™ Fixable Viability Kit, anti‐CD45, anti‐F4/80, anti‐CD11b, anti‐CD206, anti‐CD80, anti‐Thy1.2, anti‐CD4, anti‐CD8a, anti‐PD‐1, anti‐Ly6G, anti‐Ly6C and anti‐SIRPα (BioLegend). For intracellular staining, the cells were first stained for membrane‐bound protein, then treated with Fixation/Permeabilisation buffer (BD Pharmingen San Jose, California) and incubated with anti‐TNF‐α, anti‐IL‐6 and anti‐IL‐17A. Flow cytometry was performed on BD FACSAria™ III instruments (BD Biosciences, Franklin Lakes, New Jersey). During running on flow cytometry instrument, 20,000 events were recorded for each sample. Fluorescence compensation was made by single positively stained control on BD compensation beads. Data were analysed by FlowJo software, version 10.4 (Tree Star Inc.). The stained samples were carefully gated according to FMO controls (Fluorescence minus one control). Gating strategies were described in Figure S1.

### 2.5 Enzyme‐linked immunosorbent assay

Protein extracts were obtained by incubation of 50 mg tumour tissues with 500 μl RIPA buffer for 20 min (Beyotime). Protein samples were collected in supernatants after centrifuge and concentration was measured by BCA kit (Sigma), according to the manufacturer's instructions. IL‐6 and TNF‐α in tumour lysates and conditional media of treated cells were measured by enzyme‐linked immunosorbent assay (ELISA) kit, according to the manufacturer's instructions (R&D systems Inc., Minneapolis, Minnesota).

### 2.6 Western blot analysis

Protein extracts were obtained by incubation of 5 × 10^5^ BMDMs or 10^6^ LLC cells with 100 μl RIPA buffer for 20 min (Beyotime). Equal amounts of protein (25 μg/lane) were separated on sodium dodecyl sulphate‐polyacrylamide gel electrophoresis (SDS‐PAGE) and then transferred to polyvinylidene fluoride membranes (Millipore, Burlington, Massachusetts). After blocking with PBS buffer (Beyotime) containing 5% bovine serum albumin (BSA) and 0.1% Tween 20 (Beyotime), the membranes were incubated with primary antibody overnight at 4°C and followed by incubation with HRP‐conjugated secondary antibodies. The blots were finally developed with enhanced chemiluminescence (Beyotime). Antibodies used in Western blot were listed in Table S1.

### 2.7 Quantitative RT‐PCR

Total RNA was isolated by TRIzol reagent (Beyotime) following the manufacturer's instructions. cDNA was synthesised by PrimeScript RT reagent Kit with gDNA Eraser (Takara, Dalian, China). After cDNA was mixed with SYBR® Premix Ex Taq™ (TIi RNaseH Plus, Takara), a PCR reaction was performed on QuantStudio5 (Applied Biosystems). Primers used in quantitative RT‐PCR (qRT‐PCR) were listed in Table S2. β‐Actin was used as an internal loading control. Gene expression was calculated according to the 2^−△△CT^ method.

### 2.8 Immunostaining analysis

After the treated cells were fixed with 4% paraformaldehyde for 20 min and blocked with 10% goat serum and 0.1% Triton X‐100 for 30 min, primary antibodies including F4/80, p‐SHP‐1, p‐p38 MAPK, p‐STAT3 and IL‐6 were added into the cells and incubated at 4°C overnight. The cells were then incubated with FITC‐conjugated or Cy3‐conjugated second antibody for 1 h at room temperature. As for IL‐6 staining, cells were then incubated with biotin‐conjugated IL‐6 detection antibody for 1 h at room temperature, followed by incubation with PE‐Streptavidin for 1 h at room temperature. DAPI was added for nuclei staining. The stained cells were visualised under fluorescence microscope with ×200 magnification. Antibodies used for immunostaining were listed in Table S1.

### 2.9 Cell proliferation and apoptosis assay

Exactly 4 × 10^3^ LLC cells were seeded into 96‐well plates and cultured with 100 μl 50% TAM‐CM or RPMI‐1640 medium containing 5% FBS as controls. Cell proliferation was measured 0, 24, 48, 72 and 96 h after treatment by cell counting kit‐8 (Beyotime), according to the manufacturer's instructions. Cell apoptosis was analysed by PE‐Annexin V apoptosis assay kits (BD Biosciences).

### 2.10 Migration and invasion assay

LLC cell migration was measured by wound healing, LLC cells at 90% confluence in 6‐well plates were scratched with a sterile 200 μl pipette tip and then cultured with 50% TAM‐CM medium without FBS for 24 h. Cell migration was visualised under microscope with ×100 magnification. To measure LLC cell migration in the transwell plate (Corning Costar, New York, New York), LLC cells were added in upper inserts with 200 μl serum‐free medium. 50% TAM‐CM supplied with 5% FBS was added at lower chambers. After 24 h of migration, the migrated cells at lower chambers were fixed with 10% ice‐cold methanol and stained with 0.5% crystal violet before visualisation under microscope. To measure LLC cell invasion, LLC cells were seeded into Matrigel (BD Biosciences) pre‐coated plate and cultured for 24 h. Then crystal violet staining was performed for visualisation.

### 2.11 Statistical analysis

All experimental data were presented as mean ± standard deviation. Protein expression by immunostaining and Western blot was quantified by ImageJ software and followed by normalisation. Except for animal experiments, all independent experiments were conducted for three times and one representative data of three independent experiments was shown. All data were statistically analysed by GraphPad Prism 9 software. Student's *t*‐test was performed for comparison between two groups. A value of *p* < .05 was considered statistically significantly different.

## 3 RESULTS

### 3.1 Lack of SIRPα inhibited LLC cell growth in mice

**FIGURE 1:**
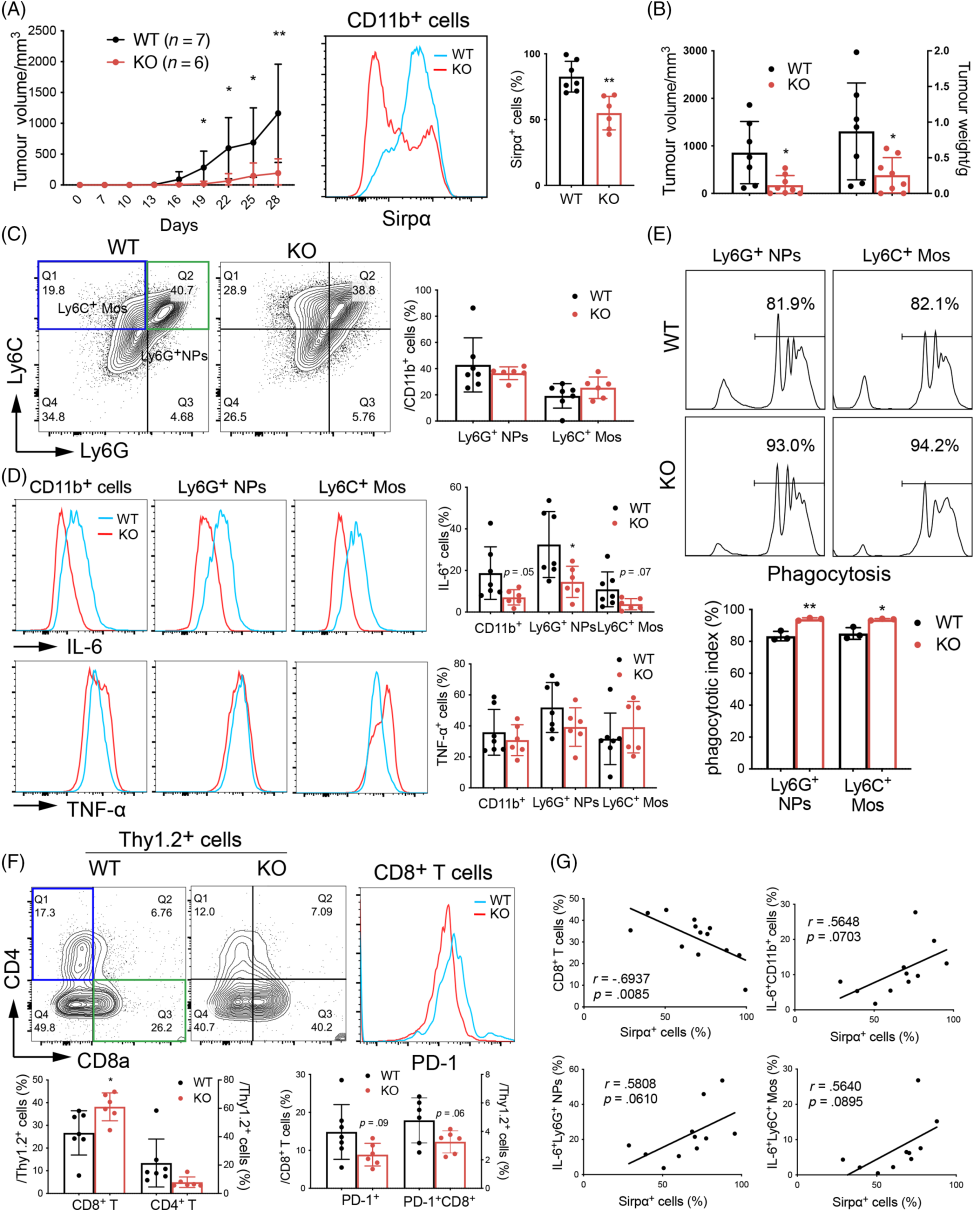
Lack of SIRPα inhibited LLC cell growth in mice. (A) The growth curve of murine lung cancer LLC cells after implantation into C57/B6 WT (*n* = 7) and SIRPα KO (*n* = 6) mice. The expression of SIRPα in CD11b^+^ cells between two groups was measured by flow cytometry and quantified. (B) The volume and weight of resected LLC tumour at the end of experiments. (C) The population of infiltrating Ly6G^+^ NPs (CD45^+^CD11b^+^Ly6G^+^Ly6C^+^) and Ly6C^+^ MPs (CD45^+^CD11b^+^Ly6G^−^Ly6C^+^) in tumour tissues of two groups were analysed by flow cytometry and quantified. (D) The expression of IL‐6 and TNF‐α in infiltrating CD11b^+^ cells, Ly6G^+^ NPs and Ly6C^+^ MPs was analysed by flow cytometry and quantified. (E) Phagocytotic activity of infiltrating Ly6G^+^ NPs and Ly6C^+^ macrophages (MPs) was analysed by flow cytometry. (F) The population of infiltrating CD8^+^ and CD4^+^ T cells and PD‐1 expression in CD8^+^ T cells was analysed by flow cytometry and quantified. (G) Correlation analysis between the population of SIRPα^+^ cells and CD8^+^ T cells, IL‐6^+^CD11b^+^ cells, IL‐6^+^Ly6G^+^ NPs and IL‐6^+^Ly6C^+^ MPs. All quantitative data were presented as mean ± standard deviation, *p < .05; **p < .01 vs. WT group, two‐tailed student *t*‐test. This experiment was conducted for three times.

As evidenced by a previous study^11^ and our study (Figure S2a), SIRPα is mainly expressed in myeloid cells, but not in T cells. We in this study found that CD47 was highly expressed on LLC cells, but the expression of SIRPα was barely detectable in LLC cells, with <10% SIRPα^+^ cells (Figure S2b). To determine the effects of SIRPα on LLC cell growth in vivo, we established a mouse tumour model by s.c. injection of LLC cells in the flank of WT and KO mice. The tumour size was measured periodically. We observed that KO mice had significantly slower tumour growth (173 mm^3^, 0.255 g), compared with those in WT mice at the end of experiments (1005 mm^3^, 0.947 g, Figure 1A,B). The SIRPα expression and infiltration of inflammatory cells were significantly reduced in CD11b^+^ myeloid cells of KO mice (Figure 1A). Though the infiltrating Ly6G^+^ NPs and Ly6C^+^ monocytes (Ly6C^+^ Mos) were comparable between the tumour tissues of WT and KO mice (Figure 1C), the expression of IL‐6 was moderately reduced in the infiltrating Ly6G^+^ NPs and Ly6C^+^ Mos (Figures 1D and S2c). However, the expression of TNF‐α was comparable between two groups. The results were further confirmed by ELISA assay, in which, there was a lower level of IL‐6 in tumour lysates of KO mice than that in WT mice (Figure S2d). To further analyse the phagocytosis activity of infiltrating NPs and Mos in mice, we purified Ly6G^+^ NPs and Ly6C^+^ Mos by flow cytometry sorting and found that infiltrating NPs and Mos in KO mice had increased phagocytosis activity than those of WT mice (Figure 1E).

Furthermore, our flow cytometry analysis showed that there were more populations of infiltrating CD8^+^ T cells in the tumour tissues of KO mice than those in WT mice, but the population of infiltrating CD4^+^ T cells was not altered. In addition, CD8^+^ T cells in KO mice expressed less immune suppressive checkpoint PD‐1 than those in WT mice (Figure 1F). The results indicated that lack of SIRPα in KO mice reduced the population of exhausted PD‐1^+^CD8^+^ T cells in tumour tissues. There was negative correlation between the population of SIRPα^+^ cells and CD8^+^ T cells, and a trend of positive correlation between the population of SIRPα^+^ cells and IL‐6^+^ cells (Figure 1G). These results confirmed the role of SIRPα in modulation of LLC cell growth in mice, in which lack of SIRPα improved NPs and Mos phagocytosis, reduced the expression of IL‐6 and infiltration of immunosuppressive PD‐1^+^CD8^+^ T cells in vivo.

### 3.2 The anti‐tumour effects of SIRPα‐KO mice were dependent on the presence of macrophages and neutrophils

**FIGURE 2:**
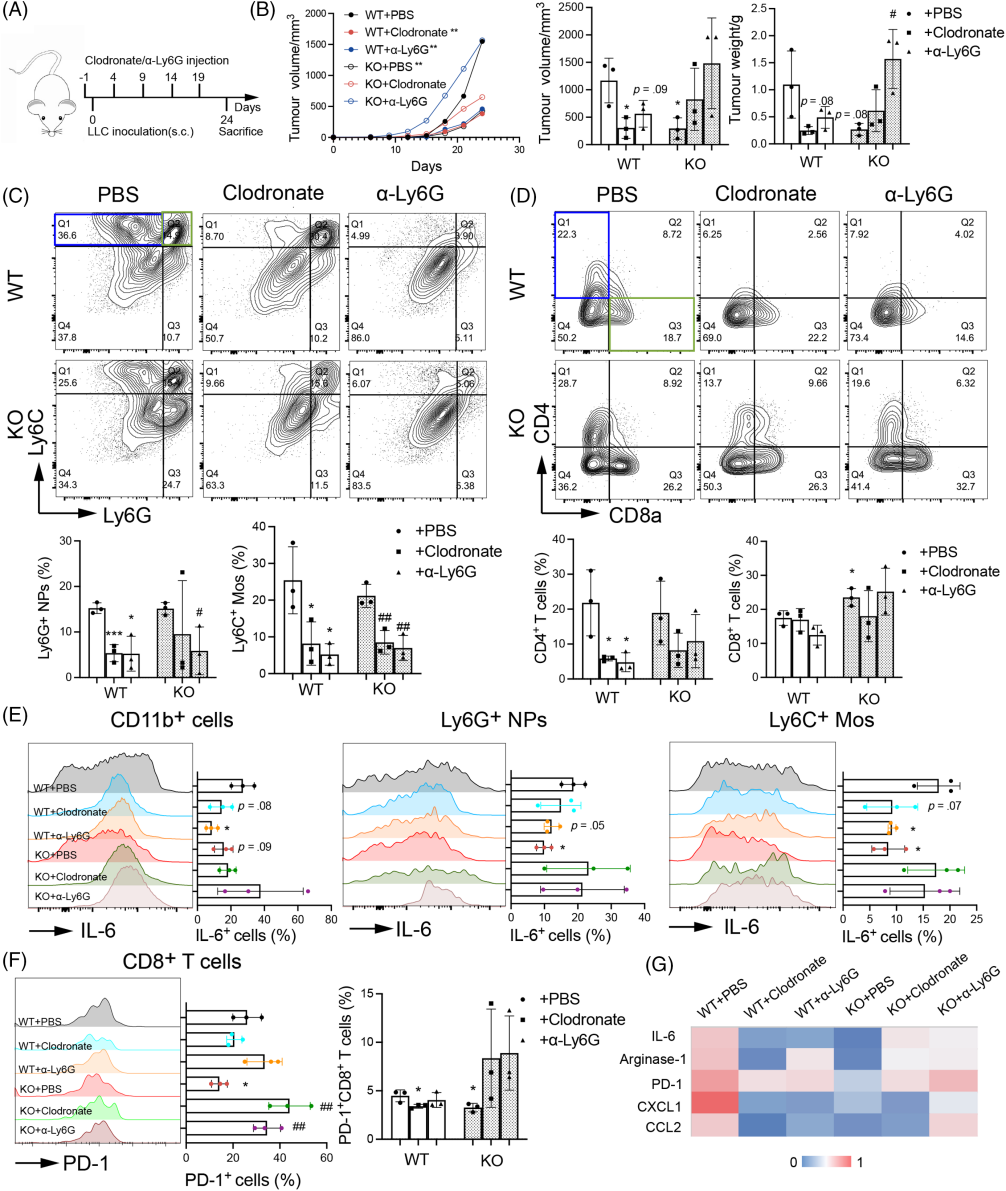
Depletion of macrophages and neutrophils abolished the anti‐tumour effects of SIRPα KO mice. (A) Schematic diagram of myeloid cell depletion and tumour engraftment in mice. (B) The growth curve of LLC cells after implantation, the volume and weight of resected tumours in six groups. Each group had three mice. (C) The population of infiltrating Ly6G^+^ NPs and Ly6C^+^ MPs was analysed by flow cytometry and quantified. (D) The population of infiltrating CD8^+^ and CD4^+^ T cells in tumour tissues was analysed by flow cytometry and quantified. (E) The expression of IL‐6 in infiltrating CD11b^+^ cells, Ly6G^+^ NPs and Ly6C^+^ MPs were analysed by flow cytometry and quantified. (F) The PD‐1 expression in infiltrating CD8^+^ T cells and the population of PD‐1^+^CD8^+^ T cells were analysed by flow cytometry and quantified. (G) qRT‐PCR analysis of mRNA transcripts in tumour tissues. Data was presented as a heatmap. All quantitative data were presented as mean ± standard deviation, *p < .05; **p < .01; ***p < .001 vs. WT + PBS group. ^#^ *p* < .05; ^##^ *p* < .01 vs. KO + PBS group; two‐tailed student *t*‐test. This experiment was conducted for two times.

To explore the role of MPs and NPs in tumour growth of SIRPα‐KO mice, we depleted these cells by i.p. injection of clodronate liposomes and anti‐Ly6G (αLy6G) antibody, respectively, 1 day before tumour inoculation of LLC cells and every 5 days afterwards (Figure 2A). Dramatically decreased MPs and NPs in blood were confirmed by flow cytometry analysis (Figure S3a), and the depletion effects were reserved at the end of animal experiments (Figure S2b). In addition, we observed that depleted of MPs and NPs lead to slower tumour growth in WT mice, compared with the WT mice without depletion of MPs and NPs (Figure 2B). Surprisingly, we observed faster tumour growth in MPs and NPs‐depleted KO mice than KO mice without depletion of MPs and NPs (Figure 2B), indicating a tumorigenic role of circulating MPs and NPs in WT mice, but an anti‐tumour role of circulating MPs and NPs in KO mice. Consistently, there were reduced infiltrating Ly6C^+^ Mos and Ly6G^+^ NPs in tumour tissues of WT and KO mice depleted of MPs and NPs, respectively (Figure 2C). Depletion of circulating MPs decreased the infiltrating Ly6G^+^ NPs in tumour tissues, and vice versa. We speculate that MPs and NPs may have mutual abilities of recruitment into tumour tissues under complicated microenvironment. Accompanied with tumour growth, the expression of IL‐6 was decreased in myeloid cell‐depleted WT mice, but was increased to some extent in myeloid cell‐depleted KO mice, compared with the nondepleted KO mice (Figure 2E).

Further studies showed that the infiltrating CD4^+^ T cells were decreased to some extent in tumour tissues of WT and KO mice depleted of MPs and NPs (Figure 2D). Though the infiltrating CD8^+^ T cells were not reduced markedly by depletion in WT and KO mice, there was reduced PD‐1 expression in the infiltrating CD8^+^ T cells of depleted WT mice. However, opposite effects were observed in depleted KO mice, in which the infiltrating CD8^+^PD‐1^+^ T cells were increased in tumour tissues of the depleted KO mice (Figure 2F). The results further indicated the distinct role of circulating MPs and NPs in WT and KO mice. In addition, we observed a reduced polarisation of CD206^+^ M2 cells and IL‐6 expression in WT mice after depletion of circulating MPs and NPs (Figure S3c,d). The results were associated with the reduced production of IL‐6, Arginase‐1, PD‐1, CXCL1 (CXC chemokine ligands 1), CCL2 (c–c motif chemokine ligand 2), which were pre‐dominantly expressed from M2 cells. However, the decreased M2 cell‐related factors tended to be reversed in myeloid cell‐depleted KO mice (Figure 2G). Thus, we conclude that SIRPα deficiency protected mice from tumour growth, dependent on the presence of MPs and NPs. MPs and NPs in TME played an important role in suppressing tumour growth of KO mice.

### 3.3 Lack of SIRPα resulted in resistance to the polarisation of M2 subtype induced by LLC cells

**FIGURE 3:**
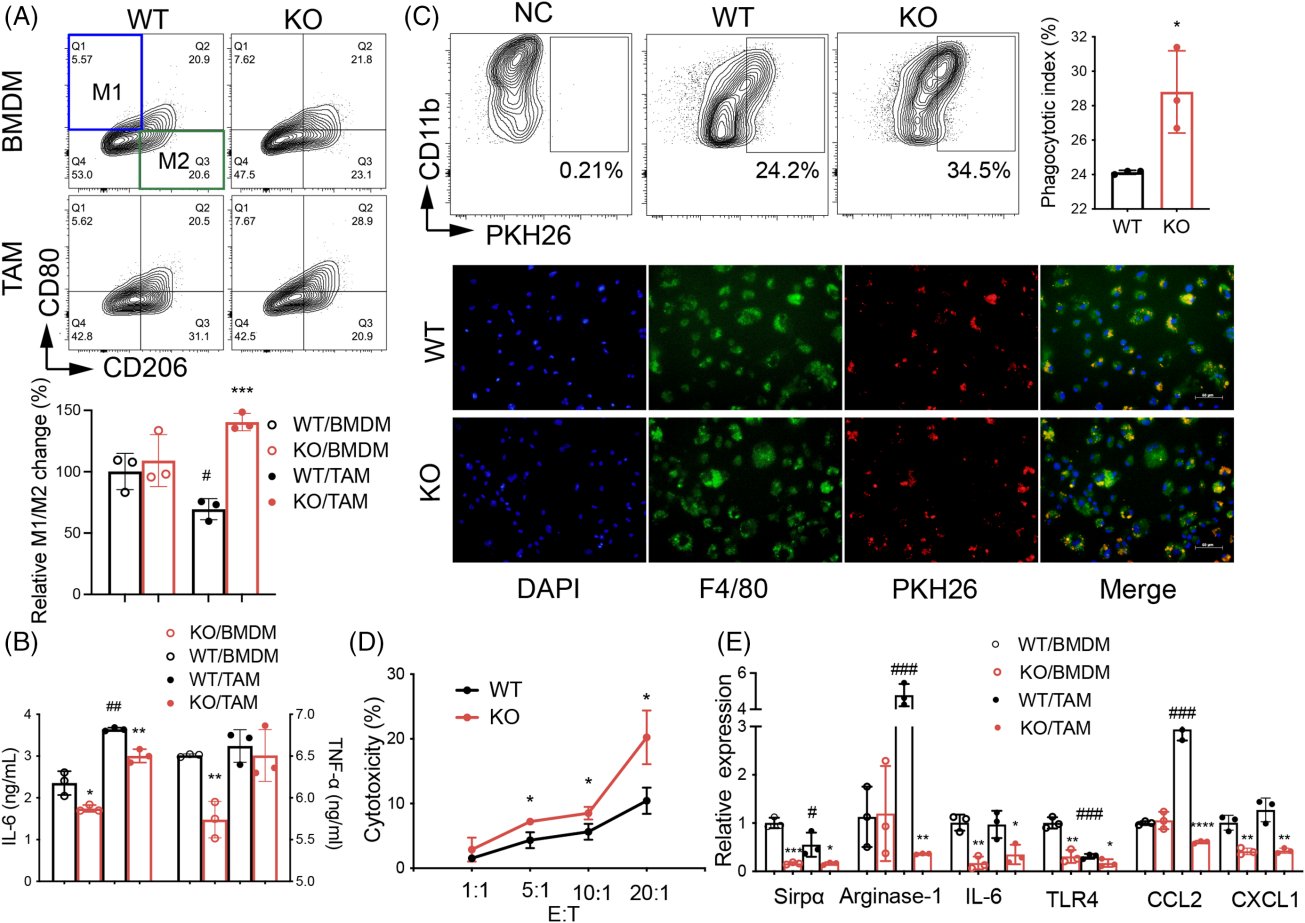
Lack of SIRPα increased the M1 subtype of macrophages. WT and KO BMDMs were untreated or treated with LLC cell conditional media to become TAMs and followed by flow cytometry analysis. (A) Representative contour plot for the expression of CD80 and CD206 (upper panel). Quantitative analysis of relative change in CD80^+^CD206^−^ M1/CD80^−^CD206^+^ M2 cell ratio (lower panel). (B) The expression of IL‐6 and TNF‐α in cell supernatants by ELISA assay. (C) Representative contour plot and quantitative analysis of WT and KO BMDM phagocytosis to PKH26‐labelled LLC cells (upper panel). Representative immunostaining images for BMDM phagocytosis. BMDMs were stained with F4/80 (lower panel). (D) Cytotoxicity of BMDMs was measured by LDH release in cell supernatants. (E) qRT‐PCR analysis for SIRPα and M2 cell relevant gene expression in the treated cells. BMDMs, bone marrow‐derived macrophages; TAMs, tumour‐associated macrophages. All quantitative data was presented as mean ± standard deviation, *p < .05; **p < .01; ***p < .001; ^****^ *p* < .0001 vs. WT controls. ^#^ *p* < .05; ^###^ *p* < .001 vs. WT/BMDM, *n* = 3, two‐tailed student *t*‐test.

To investigate the role of SIRPα expression in the modulation of macrophage phenotypes in vitro, we cultured WT and KO BMDMs respectively with LLC‐derived conditional media (LLC‐CM) for 24 h to obtain tumour‐associated macrophages (TAMs). The cells incubated with normal complete media (NC‐CM) were used as controls. The results of flow cytometry analysis showed that the percentage of M1 (CD80^+^CD206^−^) and M2 (CD80^−^CD206^+^) cells were comparable between NC‐CM incubated WT and KO BMDMs. However, LLC‐CM‐induced TAMs were featured with a reduced M1/M2 ratio (decreased to 70%), indicating the role of LLC‐CM in inducing polarisation of protumor M2 cells in vitro. Furthermore, lack of SIRPα induced resistance of KO/TAMs to LLC‐CM‐induced polarisation of M2 cells, in which the relative M1/M2 ratio was increased for up to 140% (Figure 3A).

Additionally, we observed the moderately upregulated IL‐6 in WT/TAMs, compared with the WT/BMDMs. However, the effects were reversed by the lack of SIRPα in KO/TAMs, indicating that LLC‐CM up‐regulated the expression of IL‐6 in TAMs dependent on the presence of SIRPα (Figure 3B). In consistent with results in tumour tissues, the expression of TNF‐α was comparable between KO/TAMs and WT/TAMs (Figure 3B). There were improved phagocytotic and cytotoxic activities of KO/BMDMs to LLC cells, compared with those of WT/BMDMs (Figure 3C,D). additional analysis by qRT‐PCR method showed that M2‐related gene expression, including IL‐6, Arginase‐1, Toll‐like receptor 4 (TLR4), CCL2 and CXCL1 was obviously suppressed in KO/TAMs, further confirming the role of SIRPα in promoting M2 cell polarisation (Figure 3E).

### 3.4 SIRPα reduced M1 subtype macrophage and phagocytosis through SHP‐1/p38 MAPK/STAT3 signalling

**FIGURE 4:**
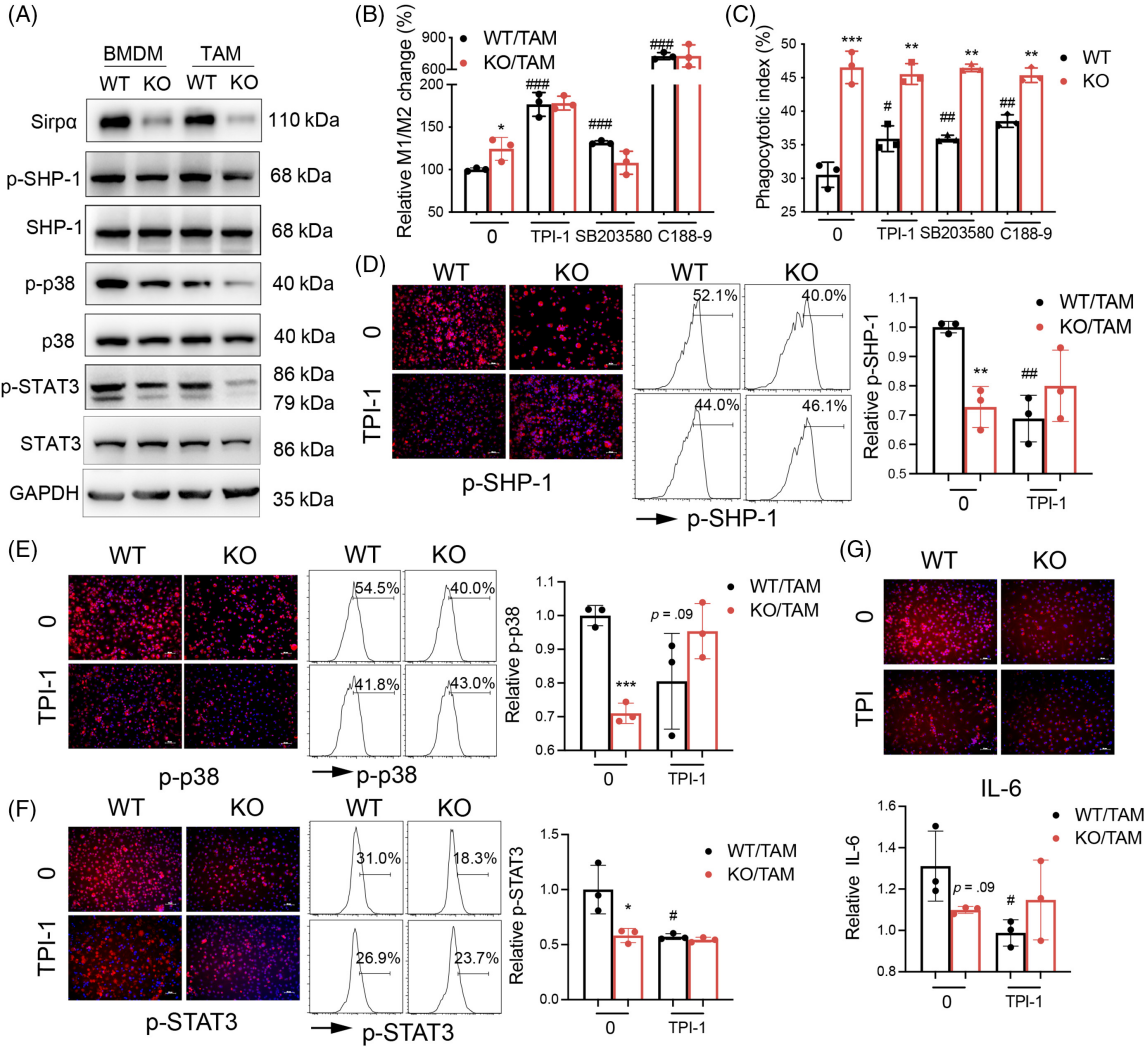
Lack of SIRPα improved macrophage phagocytosis through SHP‐1/p38 MAPK/STAT3 signalling. (A) The expression of SIRPα, p‐SHP‐1, total SHP‐1, p‐p38 MAPK, total p38 MAPK, p‐STAT3, total STAT3 and GAPDH loading control in untreated BMDMs and LLC cell conditional media‐educated TAMs were analysed by Western blot. Representative blots were shown. (B) M1 and M2 cells after treatment with SHP‐1, p38 MAPK and STAT3 inhibitors (TPI‐1, SB203580, C188‐9) were analysed by flow cytometry. The relative change in M1/M2 ratio was quantitatively analysed. (C) Quantitative analysis of inhibitor‐treated BMDM phagocytosis to PKH26‐labelled LLC cells. (D–F) Immunostaining and quantitative analysis of p‐SHP‐1, p‐p38 MAPK and p‐STAT3 in SHP‐1 inhibitor (TPI‐1)‐treated TAMs. (G) Immunostaining and quantitative analysis of IL‐6 expression in TPI‐1‐treated TAMs. Representative fluorescence images, scale bar = 50 μm. BMDMs: bone marrow‐derived macrophages; TAMs, tumour‐associated macrophages. All quantitative data was presented as mean ± standard deviation, *p < .05; **p < .01; ***p < .001 vs. WT control. ^#^ *p* < .05; ^##^ *p* < .01; ^###^ *p* < .001 vs. untreated 0/WT group, *n* = 3, two‐tailed student *t*‐test.

Previous studies demonstrated that activation of SIRPα resulted in phosphorylation of SHP‐1.^18^ In this study, we found that the lack of SIRPα in KO/TAMs decreased the expression of p‐SHP‐1, p‐p38 MAPK and p‐STAT3, compared with those in WT/TAMs. Similar results were also observed in KO/BMDMs (Figures 4A and S4a). Furthermore, SHP‐1, p38 MAPK and STAT3 inhibitors (TPI‐1, SB203580 and C188‐9) moderately increased M1 cell‐biased polarisation in WT/TAMs, with more potent effects of TPI‐1 (increased to 176%) and C188‐9 (726% increases) than SB203580 on increasing M1 cell‐biased polarisation (131% increases, Figures 4B and S4b), suggesting that lack of SIRPα increased M1‐biased polarisation and the effects were potentiated by blocking SHP‐1, p38 MAPK and STAT3 signalling in WT/TAMs. Thus, SIRPα decreased M1‐biased polarisation possibly through activation of SHP‐1/p38 MAPK/STAT3 signalling in MPs.

In addition, we observed the improved macrophage phagocytosis after treatment with TPI‐1, SB203580 and C188‐9, compared with the untreated WT/TAMs. Lack of SIRPα largely increased phagocytosis activity of KO/TAMs, with or without inhibitor treatment (Figures 4C and S4c). The results indicated a predominant role of SIRPα in suppressing macrophage phagocytosis. SHP‐1/p38 MAPK/STAT3 signalling was involved in suppressing macrophage phagocytosis as well.

To further determine the SIRPα downstream signalling pathway, WT/TAMs and KO/TAMs were treated with TPI‐1. The results of immunostaining and flow cytometry showed that suppression of SHP‐1 activity by TPI‐1 effectively suppressed p‐SHP‐1 in WT/TAMs, but not in SIRPα KO/TAMs (Figure 4D). p‐p38 MAPK and p‐STAT3 were reduced in TPI‐1 treated WT/TAMs, compared with the untreated WT/TAMs; whereas lack of SIRPα in KO/TAMs resulted in lower p‐p38 MAPK and p‐STAT3 activation than those in WT/TAMs, regardless of TPI‐1 treatments (Figure 4E,F). The results indicated the critical role of SIRPα/SHP‐1 axis in activation of p‐p38 MAPK/STAT3 signalling. In addition, we observed lower expression level of IL‐6 in TPI‐1 treated WT/TAMs, compared with the untreated WT/TAMs, and lack of SIRPα in KO/TAMs resulted in lower IL‐6 expression than the WT/TAMs, regardless of TPI‐1 treatment (Figures 4G and S4d). Therefore, SIRPα may up‐regulate IL‐6 expression through activation of SHP‐1/p38 MAPK/STAT3 signalling in MPs.

### 3.5 Lack of SIRPα on macrophages suppressed migration and invasion of LLC cells through decreasing IL‐6 activity

**FIGURE 5:**
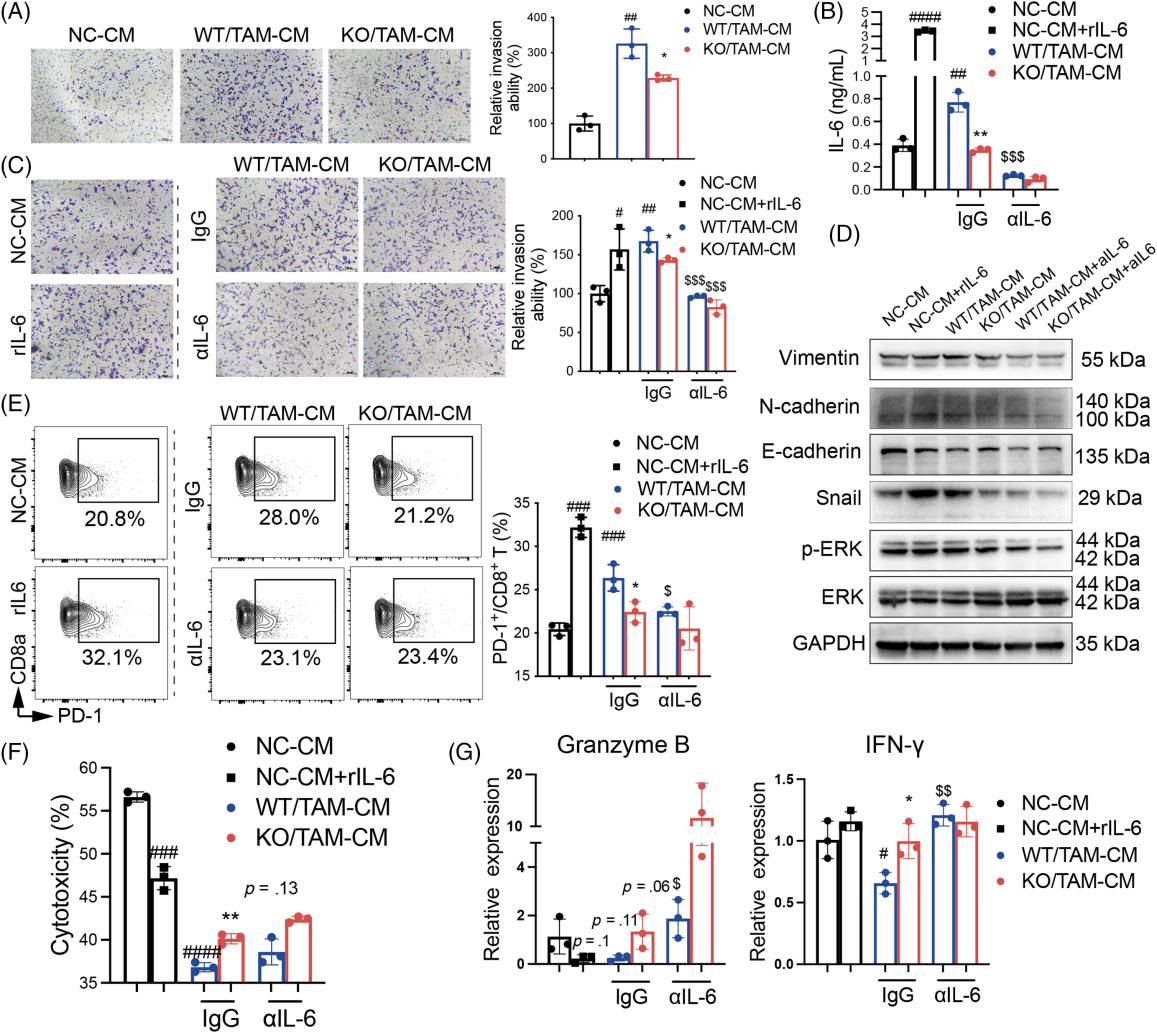
SIRPα expression in macrophages improved invasion of LLC cells through IL‐6. (A) LLC cells were incubated with 50% control NC‐CM, WT/TAM‐CM and KO/TAM‐CM medium for 24 h. LLC cell invasion was analysed by Matrigel transwell analysis. Representative invasion images (left panel) and quantitative analysis (right panel), scale bar = 100 μm. (B) ELISA analysis for the expression level of IL‐6 in supernatants of LLC cells treated with NC‐CM, WT/TAM‐CM and KO/TAM‐CM with/without pre‐neutralisation with 0.5 μg/ml anti‐IL‐6 (aIL‐6). The cells treated with NC‐CM and 10 ng/ml recombinant IL‐6 (rIL‐6) respectively were controls. (C) Invasion analysis of LLC cells treated with WT/TAM‐CM and KO/TAM‐CM with/without pre‐neutralisation with aIL‐6. Representative images (left panel), scale bar = 100 μm, and quantitative analysis (right panel). (D) Western blot analysis for EMT‐related protein in LLC cells treated with indicated CMs. Representative blots. (E) Flow cytometry analysis for the expression of PD‐1 expression in CD8^+^ T cells of splenocytes treated with indicated CMs. Representative contour plots (left panel) and quantitative analysis (right panel). (F) Cytotoxic effects were measured by LDH release assay after splenocytes were treated with indicated CMs and cocultured with LLC cells at a ratio of 20:1. (G) qRT‐PCR analysis for the expression of Granzyme B and IFN‐γ in splenocytes treated with indicated CMs. All quantitative data was presented as mean ± standard deviation, *p < .05; **p < .01; ***p < .001 vs. WT group. ^#^ *p* < .05; ^##^ *p* < .01; ^###^ *p* < .001; ^####^ *p* < .0001 vs. NC‐CM group. ^$^ *p* < .05; ^$$^ *p* < .01; ^$$$^ *p* < .001 vs. IgG group; two‐tailed student *t*‐test.

To investigate the role of SIRPα on MPs in the invasion and migration of LLC cells, we cultured LLC cells with WT and KO TAMs‐derived conditional media (TAM‐CM). The cells cultured with normal cell conditional media (NC‐CM) were controls. The results showed that WT/TAM‐CM increased LLC cell invasion and migration, compared with the cells cultured under NC‐CM controls. However, the effects were effectively reversed by KO/TAM‐CM (Figures 5A and S5a).

To determine whether IL‐6 in TAM‐CM affected epithelial‐mesenchymal transition (EMT), we measured IL‐6 levels in TAM‐CM. In line with the results before mentioned, there was an increased IL‐6 level in WT/TAM‐CM, compared with that of NC‐CM treated controls, and KO/TAM‐CM had lower IL‐6 than WT/TAM‐CM, which was neutralised by pre‐treatment with anti‐IL‐6 antibody (αIL‐6, Figure 5B). To confirm the source of IL‐6 in BMDMs, TAMs and LLC cells, we performed additional measurements, in which LLC cells produced little IL‐6 (<1 ng/ml), but BMDMs and TAMs produced higher levels of IL‐6 (over 1–10 ng/ml IL‐6) than LLC cells, indicating that BMDMs and TAMs were major sources of IL‐6 (Figure S5b). To further investigate the role of TAMs‐derived IL‐6 in LLC cells invasion and migration, we cultured LLC cells with WT/TAM‐CM and KO/TAM‐CM, respectively, with/without IL‐6 pre‐neutralisation. The results showed that LLC cells treated with 10 ng/ml recombinant IL‐6 (rIL‐6) had more invasion and migration than the NC‐CM treated cell controls, indicating the role of IL‐6 in promoting LLC cell invasion and migration. We also observed more invasion and migration of the cells treated with WT/TAM‐CM than NC‐CM, and the effects were effectively reversed by IL‐6 pre‐neutralisation in WT/TAM‐CM by αIL‐6, indicating that IL‐6 in WT/TAM‐CM promoted LLC invasion and migration. However, the LLC cells in KO/TAM‐CM had lower invasion and migration than the cells in WT/TAM‐CM. IL‐6 pre‐neutralisation reduced the ability of TAM‐CM to promote invasion and migration, regardless of SIRPα expression (Figures 5C and S5c). Furthermore, we added 5 ng/ml rIL‐6 in KO/TAM‐CM‐cultured LLC cells to determine whether rIL‐6 treatment increases LLC cells invasion and migration after cultured under KO/TAM‐CM. The results showed that rIL‐6 had no obvious effects on WT/TAMs, but partially increased ability of KO/TAMs in promoting LLC cells migration (Figure S5d) and invasion (Figure S5e). The results revealed that SIRPα‐mediated promotion in invasion and migration was partially dependent on IL‐6. We also tested EMT‐related protein expression. The results revealed that rIL‐6 and WT/TAM‐CM increased the expression of pro‐EMT protein, including Vimentin, N‐cadherin, Snail and p‐ERK1/2, compared with the cells treated with NC control. However, their expression levels were moderately reduced in the cells treated with IL‐6 pre‐neutralised WT/TAM‐CM (WT/TAM‐CM/αIL‐6) and KO/TAM‐CM with/without αIL‐6 (Figures 5D and S5f). The epithelial‐related protein E‐Cadherin had the opposite changes when LLC cells were cultured with rIL‐6 and WT/TAM‐CM, though αIL‐6 treatment could not reverse WT/TAM‐CM induced downregulation of E‐cadherin. The results demonstrated the pro‐EMT activity of WT/TAM‐CM, and the effects were dependent on IL‐6 signalling. KO/TAMs had a lower level of IL‐6 and pro‐EMT activity. Our further experiments also revealed the pro‐tumour role of TAM‐CM, in which WT/TAM‐CM promoted LLC cell proliferation (Figure S5h) and inhibited LLC cell early apoptosis, compared with the cells treated with NC‐CM control. However, the effects were comparable between LLC cells cultured in KO/TAM‐CM and WT/TAM‐CM (Figure S5g).

To investigate the effects of TAM‐CM on T cell cytotoxic activity, we treated splenocytes with WT/TAM‐CM and KO/TAM‐CM, respectively. The results revealed the increased population of exhausted CD8^+^PD‐1^+^ T cells in splenocytes treated with rIL‐6 and WT/TAM‐CM, compared with the cells treated with NC‐CM control. However, their population was moderately reduced in the cells treated with IL‐6 pre‐neutralised WT/TAM‐CM (WT/TAM‐CM/αIL‐6) and KO/TAM‐CM with/without αIL‐6, compared with the cells treated with WT/TAM‐CM (Figure 5E). The results indicated the role of IL‐6 in inducing exhausted CD8^+^PD‐1^+^ T cells in WT/TAM‐CM. LDH release analysis showed that the reduced exhaustion of CD8^+^PD‐1^+^ T cells was associated with improved cytotoxicity of CD8^+^ T cells in splenocytes of KO/TAM‐CM, compared with those of WT/TAM‐CM (Figure 5F). The effects were further supported by qRT‐PCR analysis, in which the expression of Granzyme B and IFN‐γ was increased in splenocytes of KO/TAM‐CM and IL‐6 pre‐neutralised WT/TAM‐CM (WT/TAM‐CM/aIL‐6), compared with the cells treated with WT/TAM‐CM (Figure 5G). Considering macrophages could autocrinally affect themselves by releasing mediators, we tested if IL‐6 could affect macrophage phagocytosis. Interestingly, rIL‐6 alone could not influence phagocytosis of WT/BMDMs, but decreased phagocytosis of KO/BMDMs (Figure S5i). Taken together, lack of SIRPα reduced IL‐6 expression in TAMs, subsequently attenuated migration, invasion and EMT transition of LLC cells. The anti‐tumour effects were accompanied with suppressed differentiation of exhausted CD8^+^PD‐1^+^ T cells and improved cytotoxic effects of CD8^+^ T cells.

### 3.6 SIRPα deficiency in neutrophils increased cytotoxicity to LLC cells

**FIGURE 6:**
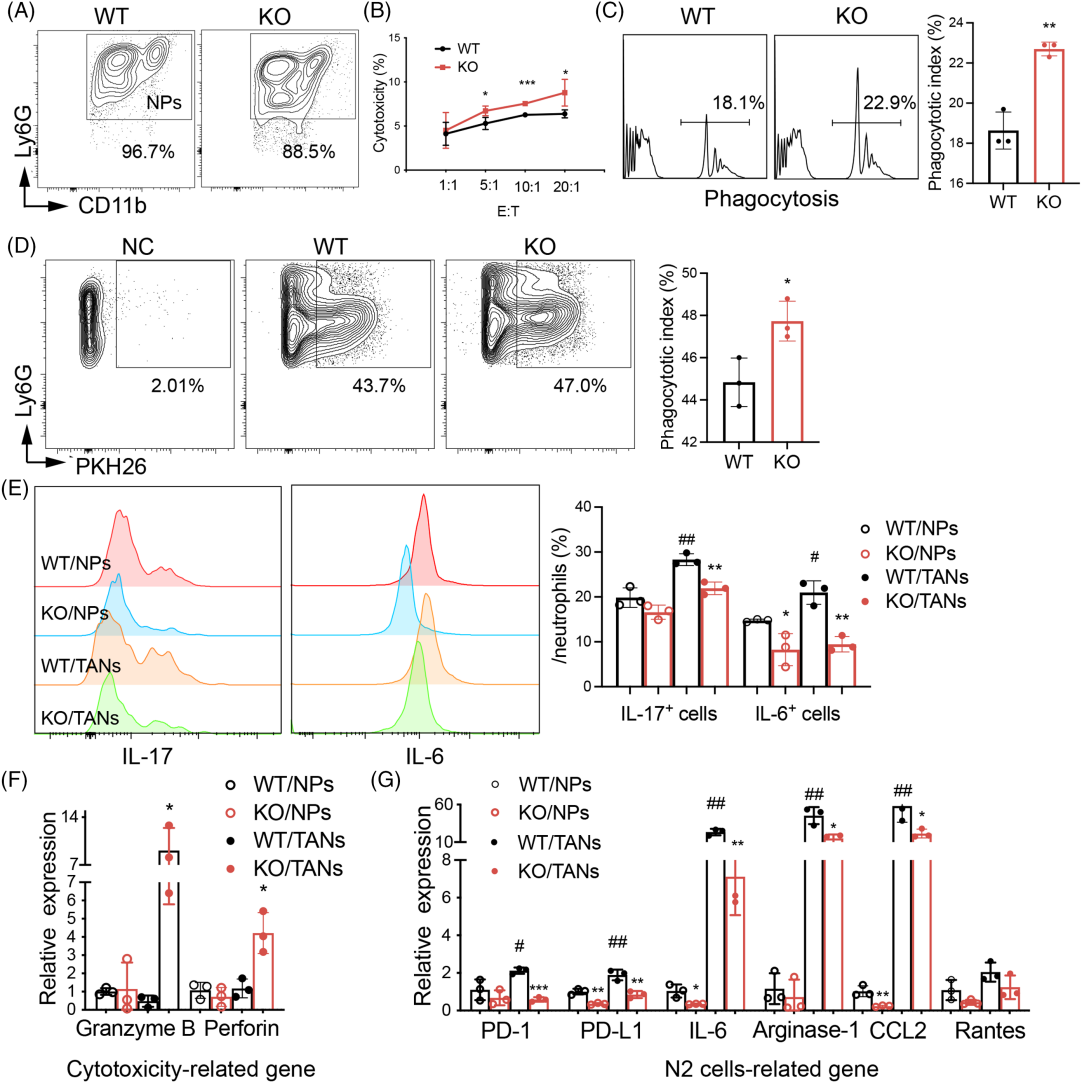
SIRPα deficiency in neutrophils increased cytotoxicity to LLC cells. (A) NPs were collected from the bone marrow of WT and KO mice, and purity was analysed by flow cytometry. (B) WT and KO NPs were co‐cultured with LLC cells at indicated ratio (E:T, NPs:LLC cells). Cytotoxicity was determined by LDH release in cell supernatants. Phagocytosis of NPs was analysed by flow cytometry after incubation with beads (C, left panel) and PKH26^+^ LLC cells (D, left panel), respectively. NC, Target cells were added to control. The phagocytosis index was quantified (right panel). (E) The expression of IL‐17 and IL‐6 in NPs and TANs (LLC‐CM treated NPs) were analysed by intracellular staining and flow cytometry. Representative histogram (left panel). The percentage of positively stained cells was statistically analysed (right panel). (F) qRT‐PCR analysis of cytotoxicity‐related genes in purified NPs and TANs. (G) qRT‐PCR analysis of N2 cell‐related genes. Data were presented as 2^−(ΔΔct)^ relative to internal GAPDH control. NPs: neutrophils; TANs: tumour‐associated neutrophils. All quantitative data was presented as mean ± standard deviation, *p < .05; **p < .01; ***p < .001 vs. WT group. ^#^ *p* < .05; ^##^ *p* < .01 vs. WT NPs group; two‐tailed student *t*‐test.

Neutrophils are heterogeneous myeloid cells with anti‐ and pro‐tumour properties, playing an important role in tumour progression at different stages.^25^, ^26^ TANs also could be clarified into N1 and N2 according to the M1 and M2 paradigm; skewing toward N2 was characterised by high expression of CCL2, CXCL1 and Arginase‐1.^27^, ^28^ To explore the biological function of SIRPα in TANs in vitro, we purified NPs from the bone marrow of WT and KO mice by Percoll gradient centrifugation, more than 80% purity of NPs was obtained (Figure 6A). After incubation of NPs with fluorescence‐labelled beads or LLC cells, more phagocytotic effects in KO/NPs were observed than those in WT/NPs (Figure 6B–D). Consistent with the results of TAMs in vitro, LLC‐CM educated WT/NPs (WT/TANs) expressed more IL‐17 and IL‐6 than that uneducated WT/NPs, which was effectively reversed by the lack of SIRPα expression in KO/TANs (Figure 6E), indicating that the increased IL‐17 and IL‐6 in TANs were dependent on SIRPα signalling. The expression of cell cytotoxicity‐related genes, including Granzyme B and Perforin, was increased in KO/TANs, compared with those in WT/TANs (Figure 6F). In contrast, N2 cell‐related genes including PD‐1, programmed cell death 1 ligand (PD‐L1), IL‐6, Arginase‐1, CCL2 and RANTES were downregulated in KO/TANs, compared with those in WT/TANs (Figure 6G). Thereby, similar to the role of SIRPα in TAMs, lack of SIRPα in TANs increased phagocytosis activity of TANs, associated with reduced expression of IL‐17, IL‐6 and N2 cell‐related genes.

### 3.7 GEPIA analysis of SIRPα expression in human lung cancer

Finally, we evaluated the characteristics of SIRPα expression in human lung cancer by gene expression profiling interactive analysis database (GEPIA). Patients with higher expression of SIRPα had worse disease‐free survival (DFS) and overall survival (OS) in lung squamous cell carcinoma (LUSC), but SIRPα had no predictive value in lung adenocarcinoma (LUAD, Figure S6a,b). In addition, the level of SIRPα was positively correlated to these cancer‐related immune suppressive checkpoints, including PD‐1 (pdcd1), PD‐L1 (cd274), CTLA‐4 (ctla‐4) and TIM‐3 (havcr2) in patients with LUAD and LUSC (Figure S6c). In addition, there were positive correlations between SIRPα and other chemokines and chemokine receptors, such as CCR2 (*ccr2*), CCL2 (*ccl2*) and CSF1R (*csf1r*) in patients with LUAD and LUSC (Figure S6d). Other moderately positive correlations including IL‐6, TNF‐α and TGF‐β were also observed (Figure S6e). Therefore, high expression of these molecules predicts a poor prognosis, and SIRPα could be a promising prognosis biomarker in patients with lung cancer.

## 4 DISCUSSION

Accumulating researches had shed light on the synergistic effects of SIRPα blockade and other agents, such as tumour‐associated antigens and immune checkpoint inhibitors.^6^, ^16^ We in this study for the first time, explored the effects of SIRPα alone on lung tumour cell growth in vivo and in vitro. The results confirmed that the lack of SIRPα in KO mice significantly reduced LLC tumour cell growth in mice. Increased phagocytosis of infiltrating NPs and MPs, decreased LLC cell migration and invasion, attenuated IL‐6 production and T cell exhaustion were believed to be involved in the antitumor activity in KO mice through SHP‐1/p38 MAPK/STAT3 signalling. The results supported the previous report, in which blockade of SIRPα signalling effectively reduced tumour growth in other animal models and overcame resistance to ICIs.^6^

CD11b^+^Gr‐1^+^ myeloid‐derived suppressor cells (MDSCs) are immature cells with immunosuppressive properties in TME. MDSCs consist of two subsets, Ly6G^+^Ly6C^low^ NPs and Ly6G^−^Ly6C^+^ Mos, which are viewed as the precursors of TANs and TAMs, respectively.^29^ We did not use anti‐Gr‐1 antibody to analyse MDSC cell subsets. Instead, Ly6G^+^ NPs and Ly6C^+^ Mos was used to identify two major subsets of myeloid cells. In this study, we observed that depletion of MPs and NPs inhibited tumour growth in WT mice, that was in consistent with the previous reports.^26^, ^30^ However, depletion of MPs and NPs abolished the suppressive effects on tumour growth in KO mice, indicating an antitumor role of circulating MPs and NPs in lung tumour growth of KO mice.

Emerging evidence showed that NPs and MPs could improve tumour initiation and progression in WT mice via crosstalk. NPs can recruit MPs and regulatory T cells (Treg) into tumour tissues by releasing CCL2 and CCL17.^31^ On the other hand, MPs can induce NP migration by releasing some neutrophil chemokine attractants, such as CXCL1 and CXCL2.^32^, ^33^ Thereby, MPs and NPs interacted with each other, and depletion of circulating MPs or NPs reduced the infiltration of pro‐tumour MPs or NPs in tumour tissues and subsequently reduced tumour growth in WT mice.

However, myeloid cell phenotypes and proportion were spatially and temporally changed during tumour progression, presenting distinct biological functions. Infiltrating NPs were predominately presented in the early stage of tumour, and replaced by MPs at the later stage of tumour, which accounts for 50% of tumour mass.^28^, ^34^ Because of the complexed immune suppressive tumour environment, the infiltrating NPs and MPs are gradually polarised from pro‐inflammatory M1 and N1 to anti‐inflammatory M2 and N2 cells, so‐called TAMs and TANs.^27^, ^35^ In contrast, NPs and MPs of KO mice had increased phagocytosis activity and exert potent antitumor effects, because we observed the reduced lung tumour growth in KO mice, which was effectively abolished in KO mice depleted of NPs and MPs. The results were consistent with the previous report, in which blockade of TGF‐β attenuated tumour growth with increased activation of infiltrating CD8^+^ T cells and anti‐tumour CD11b^+^Ly6G^+^ TANs. However, depletion of NPs blunted the antitumor effects after TGF‐β signalling was blocked.^26^ Thus, we conclude that lack of SIRPα reduced lung tumour growth in KO mice, dependent on the presence of antitumor NPs and MPs.

It was documented that MPs and NPs are major sources of IL‐6. High expression of IL‐6 was discovered in many tumour tissues and positively correlated to poor prognosis of cancer.^7^, ^9^ Thus, IL‐6 may play an important role in tumour progression. In this study, we observed that the anti‐tumour effects of KO mice were closely associated with IL‐6 expression from NPs and MPs. We speculate that the reduced IL‐6 expression may account for anti‐tumour effects in KO mice. Indeed, our further in vitro analysis confirmed that blockade of IL‐6 activity in TAMs effectively reduced LLC cell migration, invasion and EMT, meanwhile increased CD8^+^ T cell cytotoxic effects. The results further confirmed the protumor role of IL‐6 in tumour progression. The results were also supported by previous study, in which IL‐6 expression level in cancer patients was considered as a predictor of poor prognosis.^9^ There are distinct roles of IL‐6 in modulation of tumour growth. On the one hand, IL‐6 is predominantly expressed by M1 cells in the early phase of inflammation and tumour development, having pro‐inflammatory and anti‐tumour effects.^36^ On the other hand, IL‐6 is predominantly expressed by M2 cells in the later phase of inflammation and tumour development, it exerted anti‐inflammatory and pro‐tumour effects, respectively.^4^, ^7^, ^37^ Despite the potent protumor role of IL‐6, we in this study found that rIL‐6 was unable to completely restore the suppressive effects of KO/TAMs in LLC cells invasion and migration. Though neutralisation of IL‐6 in TAM/CM could increase the expression of Granzyme B and IFN‐γ, implying the role of IL‐6 in suppressing Granzyme B and IFN‐γ expression, addition of rIL‐6 alone failed to completely inhibit their expression in splenocytes as observed in this study. We speculated that their expression maybe influenced by other mediators, such as IL‐10^38^ and TLR2.^39^ Additionally, we found that the improved phagocytosis of KO/BMDMs was inhibited by addition of rIL‐6, that confirmed the role of IL‐6 expression in suppressing phagocytosis. The concept will be further investigated in the future. Furthermore, our in vitro study confirmed the role of STAT3 signalling in promoting polarisation of M2 cells, in which lack of SIRPα in MPs reduced the activation of STAT3 and expression of IL‐6, meanwhile increased the M1/M2 cell ratio. Thereby, we concluded that the lack of SIRPα exerted antitumor effects possibly by increasing the M1/M2 cell ratio, decreasing the IL‐6 secretion and suppressing STAT3 signalling.

We also found that lack of SIRPα increased phagocytosis activity of MPs and NPs in vitro. Suppression of STAT3 activity by STAT3 inhibitor, C188‐9 further improved the phagocytosis ability. Thus, we thought that lack of SIRPα exerted anti‐tumour effects possibly by increasing phagocytosis activity of MPs and NPs by suppressing STAT3 signalling. On the other hand, SIRPα suppressed phagocytosis activity of MPs through activation of STAT3 signalling, consistent with previous reports in which STAT3 signalling was negatively correlated to macrophage phagocytosis.^40^, ^41^, ^42^ However, a positive correlation between macrophage phagocytosis and STAT3 signalling was previously reported.^20^, ^43^ The controversial role of STAT3 signalling in macrophage phagocytosis will be clarified in the future.

In addition to the reduced activation of STAT3, we found that activation of SHP‐1 and p38 MAPK signalling was also reduced in KO mice. It is known that SHP‐1 is activated by SIRPα, participating in the dephosphorylation of p‐STAT3 and p‐p38 MAPK, due to its phosphatase activity.^44^ Thus, SIRPα theoretically suppressed activation of STAT3 and p38 MAPK through SHP‐1 activation, subsequently reducing macrophage phagocytosis activity. Accordingly, STAT3 and p38 MAPK signalling have a role in promoting macrophage phagocytosis as previously reported.^20^, ^45^ However, we in this study, observed reduced p38 MAPK activation and improved phagocytosis in KO mice, indicating the suppressive role of p38 MAPK signalling in macrophage phagocytosis, which is not reported previously. The controversy will be clarified in our future studies.

It is noted that there are some limitations in this study. First, we depleted neutrophils and macrophages before LLC cells inoculation, which did not completely mimic patients who have already developed tumour. Thus, depletion of neutrophils and macrophages after tumour growth in mice would be more mimic to clinical status, and the issue will be addressed in our future experiments. Second, the effects of SIRPα on lung metastases was not evaluated in this study due to short‐time course of animal experiments in this study. A longer time course of animal study will be performed to evaluate the effects of SIRPα on lung metastases in KO mice in the future. Third, we only analysed the expression of PD‐1 in CD8^+^ T cells, the expression of IFN‐γ, Granzyme B and Perforin should be analysed in CD8^+^ T cells to investigate the effects of SIRPα on CD8^+^ T cells cytotoxicity in the future.

**FIGURE 7:**
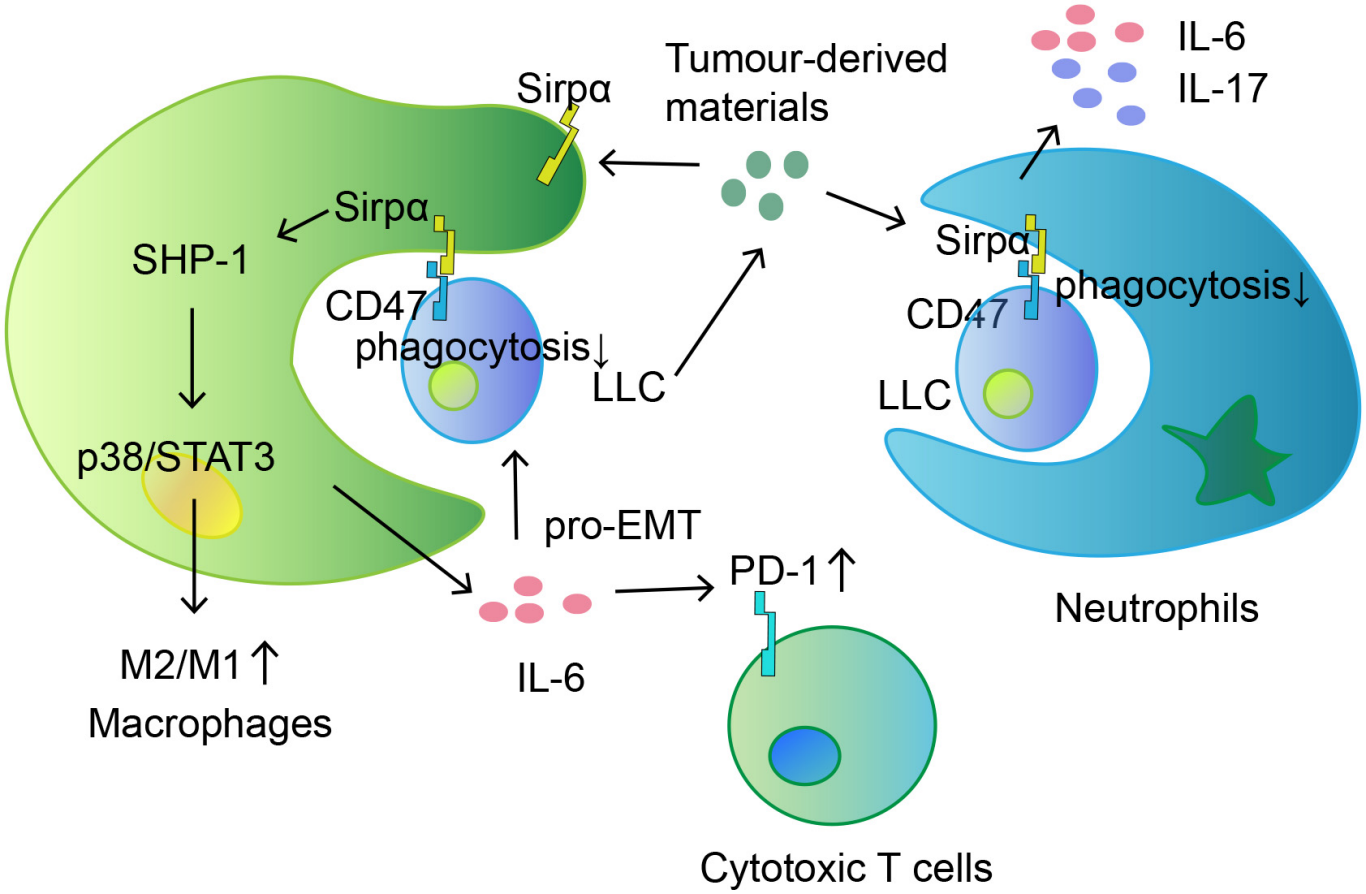
Graphical abstract for SIRPα‐mediated mechanisms in the tumour microenvironment. SIRPα inhibits MPs and NPs phagocytosis. Tumour‐derived mediators induce M2 cell polarisation and IL‐6 expression in MPs and NPs through SIRPα/SHP‐1/p38 MAPK/STAT3 signalling. IL‐6 induces epithelial–mesenchymal transition (EMT) of LLC cells and PD‐1 expression in CD8^+^ T cells. MPs, macrophages; NPs, neutrophils.

Taken together, we in this study conclude that lack of SIRPα attenuated lung cancer growth in mice by reducing SHP‐1/STAT3/p38 MAPK signalling, subsequently suppressing IL‐6 expression, meanwhile improving M1 and N1 subtypes and their phagocytosis activity (Figure 7). SIRPα could be a useful target in immunotherapy of lung cancer.

## AUTHOR CONTRIBUTIONS

Linyue Pan participated in cell culture, ELISA analysis, animal experiments, flow cytometry, data analysis and assembly and manuscript writing. Bin Wang, Mengjie Chen, Yuan Ma and Bo Cui participated in cell culture, Western blot, PCR and data analysis. Zhihong Chen, Yuanlin Song and Lijuan Hu generated hypotheses and interpreted the data. Zhilong Jiang conceived the project, revised the manuscript and was responsible for all directions of the work. All authors read and approved the final manuscript.

## CONFLICT OF INTEREST

The authors declare no competing interests.

## Supporting information


**Figure S1.** Gating strategies of flow cytometry. Gating strategy for T cells (a), myeloid cells, TNF‐α^+^ and IL‐6^+^ cells (b) and M2 cells (c) in tumour digests. Gating strategy for MPs and NPs in the blood (d), CD8^+^ T cells and PD‐1^+^ cells in splenocytes (e) and NPs extracted from bone marrow cells (f). FMO, fluorescence minus one; MPs, macrophages; NPs, neutrophils.
**Figure S2.** Lack of SIRPα reduced the expression of IL‐6 and TNF‐α in NPs and Mo in mice. (a) Flow cytometry for CD3^+^ T cells, Ly6G^+^ NPs and Ly6C^+^ MPs in blood and SIRPα expression in splenocytes between WT and KO mice. (b) Flow cytometry for CD47 and SIRPα expression in LLC cells. (c) Median fluorescence intensity (MFI) of IL‐6 and TNF‐α expression in CD11b^+^ cells, Ly6G^+^ NPs and Ly6C^+^ MPs was analysed by flow cytometry. (d) ELISA assay for IL‐6 and TNF‐α expression levels in tumour lysates. Mo, monocytes; NPs, neutrophils. Data were presented as mean ± standard deviation. Two‐tailed student *t*‐test.
**Figure S3.** Depletion of circulating monocytes and NPs by Clodronate and αLy6G reduced M2 cells and IL‐6 in mice. (a) Depletion of CD11b^+^CD11c^+^ MPs and CD11b^+^Ly6G^+^ NPs in the blood of mice before (a) and at the end of experiments (b) after treatment with Clodronate and anti‐Ly6G antibody (αLy6G). Representative contour plots of flow cytometry (upper panel). The percentage of NPs and MPs was quantitatively analysed (lower panel). (c) Flow cytometry for tumour‐infiltrating CD206^+^ M2 cells (left panel) and the percentage of M2 cells were quantified (right panel). (d) ELISA assay for IL‐6 expression in tumour lysates. MPs: macrophages; NPs, neutrophils. All quantitative data was presented as mean ± standard deviation, *p < .05; **p < .01 vs. WT + PBS group. ^#^ *p* < .05; ^##^ *p* < .01 vs. KO + PBS group; two‐tailed student *t*‐test.
**Figure S4.** Lack of SIRPα reduced activation of SHP‐1, p38 MAPK and STAT3 in BMDMs and TAMs. (a) Quantitative analysis of Western blot results for the expression of SIRPα, p‐SHP‐1, p‐p38 MAPK and p‐STAT3 in WT and KO BMDMs untreated or treated with LLC‐CM (TAMs). Data was presented as a ratio to GAPDH loading control or total protein. (b) Representative flow cytometry for CD80^+^CD206^−^ M1/CD80^−^CD206^+^ M2 cells in WT and KO TAMs treated with/without inhibitors. (c) Representative flow cytometry for phagocytosis of WT and KO TAMs treated with/without inhibitors. Target cells were PKH26‐labelled LLC cells. (d) ELISA analysis for the expression level of IL‐6 in supernatants of WT and KO BMDMs treated with LLC‐CM (TAMs, left panel) and LLC cells (cell–cell contact culture, right panel), with/without the addition of inhibitors. BMDMs: bone marrow‐derived macrophages; TAMs, tumour‐associated macrophages. Data were presented as mean ± standard deviation, *p < .05; **p < .01; ***p < .001; ^****^ *p* < .0001 vs. WT cells. ^#^ *p* < .05; ^##^ *p* < .01; ^####^ *p* < .0001 vs. NC‐CM; two‐tailed student *t*‐test.
**Figure S5.** TAM‐CM increased LLC cell migration is dependent on IL‐6. (a) Representative images of LLC cell migration and quantitative analysis after the cells were cultured in WT and KO TAM‐CM. (b) ELISA analysis for IL‐6 expression level in the supernatants of LLC cells, BMDMs cultured alone or in the indicated medium and cells. (c) Representative images of LLC cell invasion and quantitative analysis after the cells were cultured in the indicated medium pre‐treated with/without αIL‐6. (d,e) Representative images of LLC cell migration (d), invasion (e) and quantitative analysis after the cells were cultured in the indicated medium with/without rIL‐6. (f) Quantitative analysis for Western blot results. Data was ratio to internal loading control. (g) Flow cytometry analysis for LLC cell apoptosis cultured in WT and KO TAM‐CM. (h) The proliferation of LLC cells cultured in WT and KO TAM‐CM was analysed by CCK‐8. (i) Representative flow cytometry for phagocytosis of WT and KO BMDMs treated with/without rIL‐6. NC‐CM: normal cell conditioned medium; TAM, tumour‐associated macrophages. All quantitative data was presented as mean ± standard deviation, *p < .05; **p < .01; ***p < .001; ^****^ *p* < .0001 vs. WT group. ^#^ *p* < .05, ^##^ *p* < .01; ^###^ *p* < .001; ^####^ *p* < .0001 vs. NC‐CM. ^$^ *p* < .05; ^$$^ *p* < .01 vs. IgG group or Control group; two‐tailed student *t*‐test.
**Figure S6.** Characteristics of human SIRPα expression analysed by GEPIA. (a,b) Overall survival and disease‐free survival in patients with lung squamous cell carcinoma (LUSC) and lung adenocarcinoma (LUAD). (c,d) Association between SIRPα expression and immune checkpoints and chemokines in patients with LUAD and LUSC. (e) Association between SIRPα expression and cytokines in patients with LUAD and LUSC. GEPIA, gene expression profiling interactive analysis database.

**Table S1.** Information of antibodies used for western blot (WB), immunofluorescence (IF) and enzyme‐linked immunosorbent assay (ELISA).
**Table S2.** Primers used in manuscript.

## Data Availability

All data and materials were available from the corresponding author.
