# Supplementary material for: Lack of SIRP‐alpha reduces lung cancer growth in mice by promoting anti‐tumour ability of macrophages and neutrophils
Source: Cell Prolif. 2022 Nov 23;56(2):e13361. doi: 10.1111/cpr.13361 (PMC9890530; doi:10.1111/cpr.13361)

Supplemental Figure 1


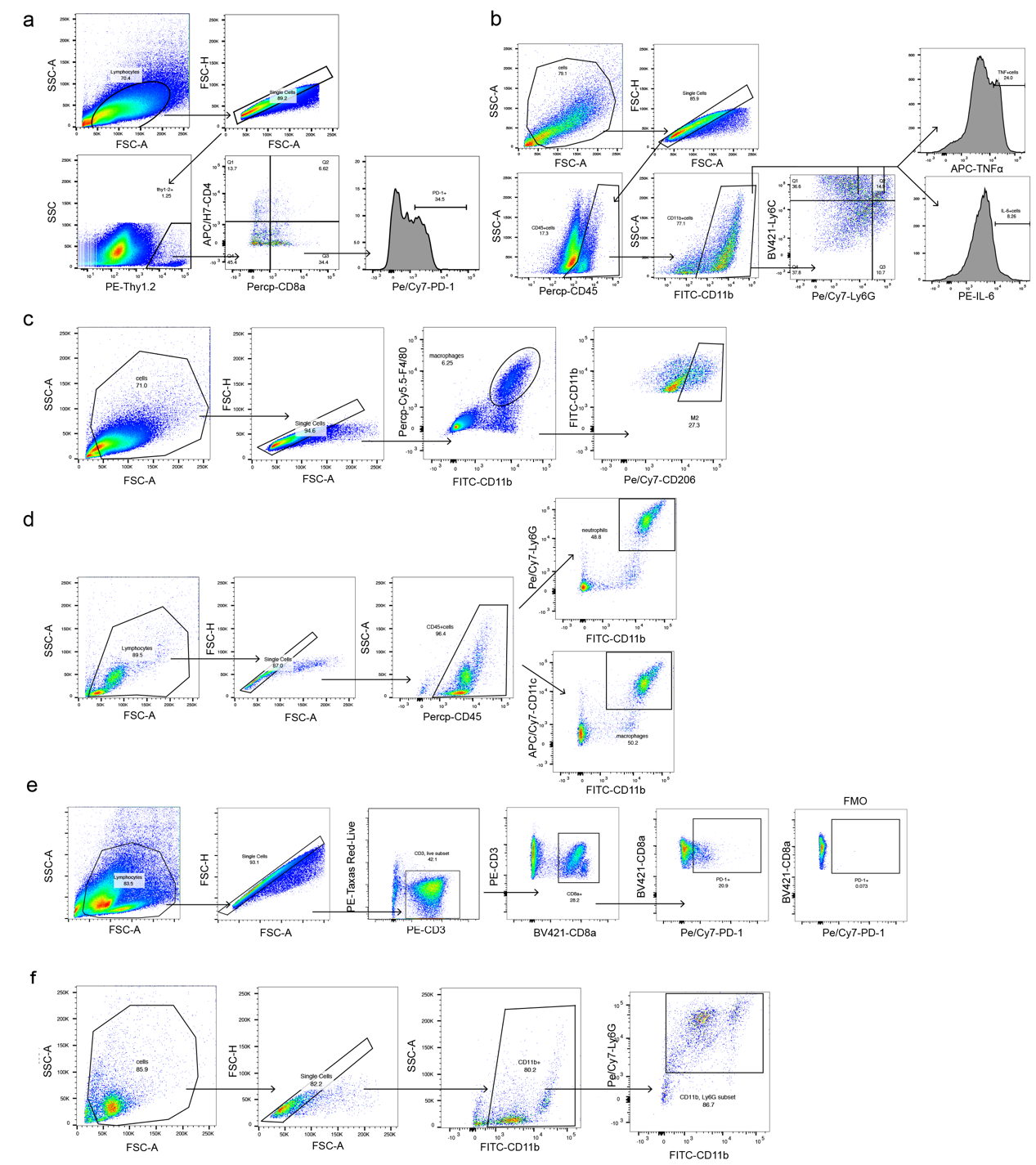


Supplemental Figure 2


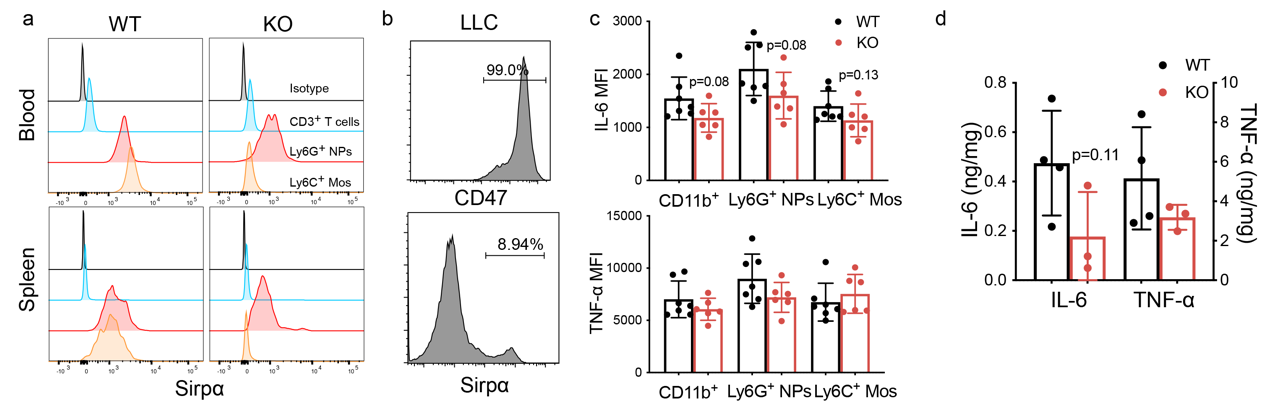


Supplemental Figure 3


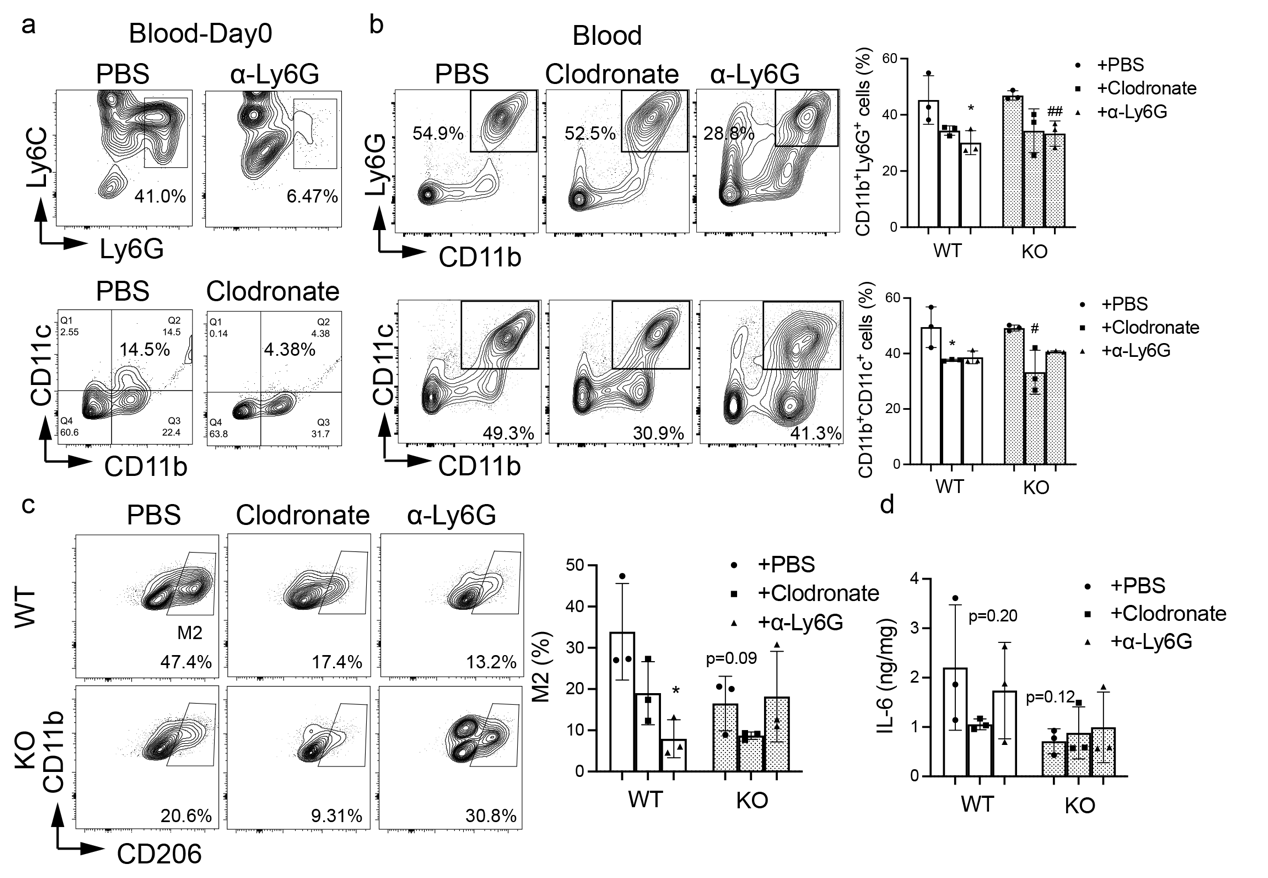


Supplemental Figure 4


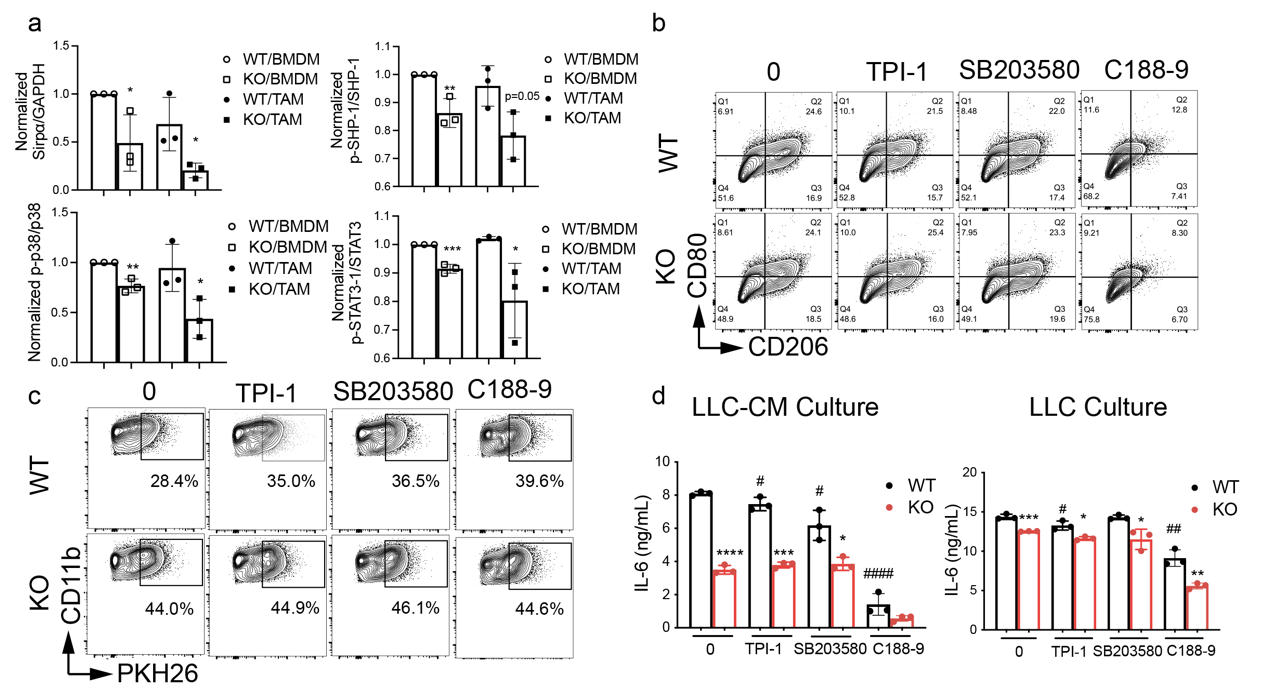


Supplemental Figure 5


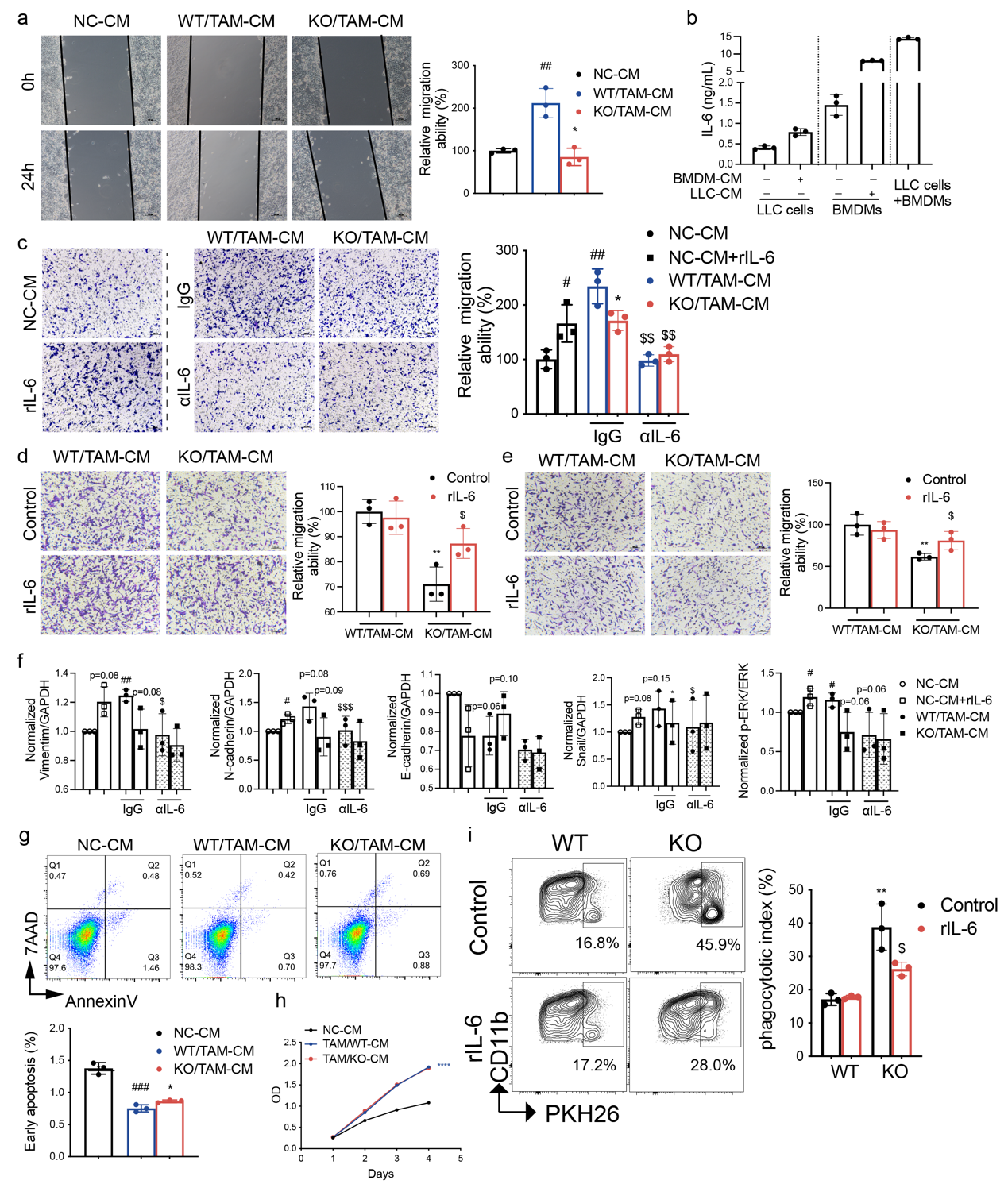


Supplemental Figure 6


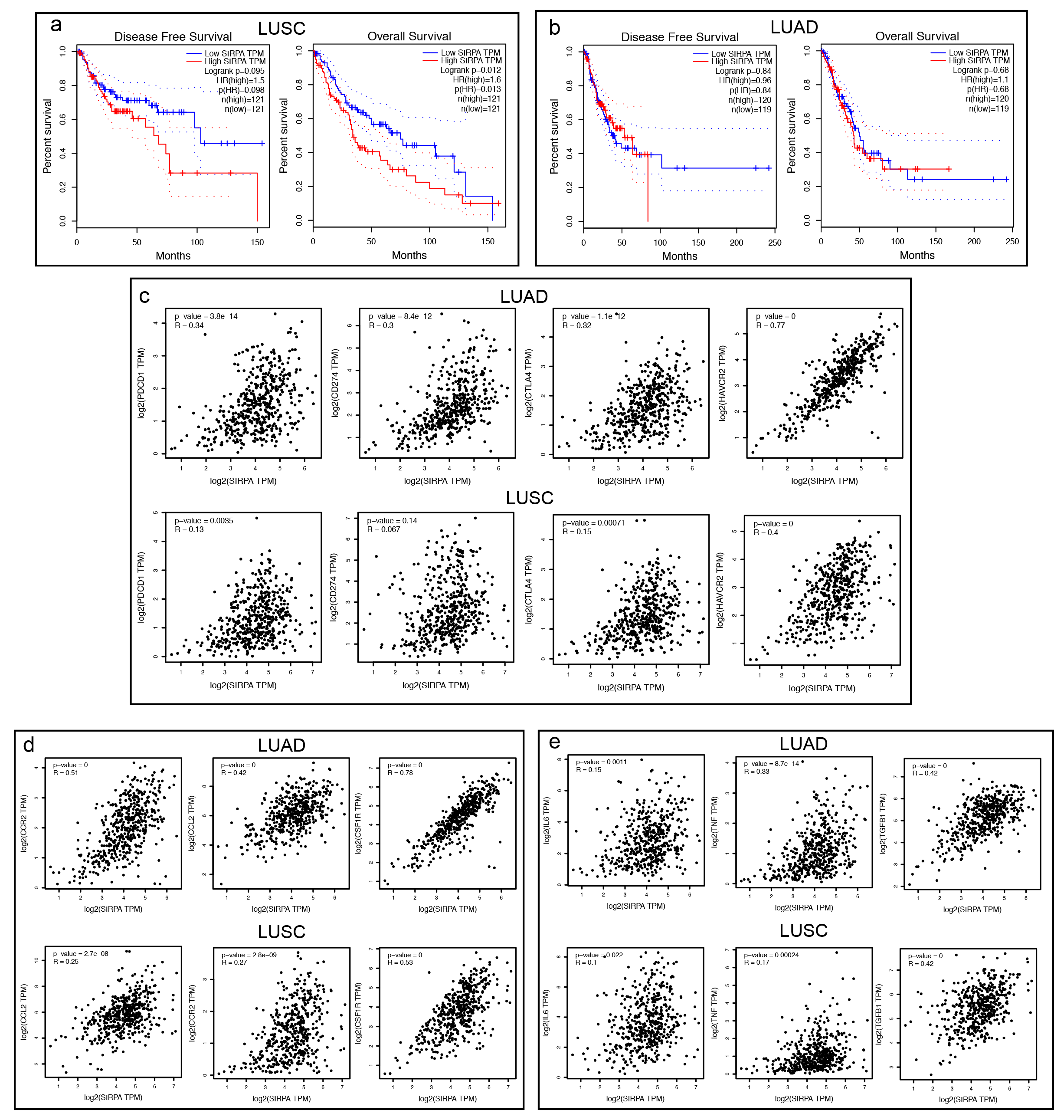

Supplement: Supplementary file 1 — Figure S1. Gating strategies of flow cytometry. Gating strategy for T cells (a), myeloid cells, TNF‐α+ and IL‐6+ cells (b) and M2 cells (c) in tumour digests. Gating strategy for MPs and NPs in the blood (d), CD8+ T cells and PD‐1+ cells in splenocytes (e) and NPs extracted from bone marrow cells (f). FMO, fluorescence minus one; MPs, macrophages; NPs, neutrophils. Figure S2. Lack of SIRPα reduced the expression of IL‐6 and TNF‐α in NPs and Mo in mice. (a) Flow cytometry for CD3+ T cells, Ly6G+ NPs and Ly6C+ MPs in blood and SIRPα expression in splenocytes between WT and KO mice. (b) Flow cytometry for CD47 and SIRPα expression in LLC cells. (c) Median fluorescence intensity (MFI) of IL‐6 and TNF‐α expression in CD11b+ cells, Ly6G+ NPs and Ly6C+ MPs was analysed by flow cytometry. (d) ELISA assay for IL‐6 and TNF‐α expression levels in tumour lysates. Mo, monocytes; NPs, neutrophils. Data were presented as mean ± standard deviation. Two‐tailed student t‐test. Figure S3. Depletion of circulating monocytes and NPs by Clodronate and αLy6G reduced M2 cells and IL‐6 in mice. (a) Depletion of CD11b+CD11c+ MPs and CD11b+Ly6G+ NPs in the blood of mice before (a) and at the end of experiments (b) after treatment with Clodronate and anti‐Ly6G antibody (αLy6G). Representative contour plots of flow cytometry (upper panel). The percentage of NPs and MPs was quantitatively analysed (lower panel). (c) Flow cytometry for tumour‐infiltrating CD206+ M2 cells (left panel) and the percentage of M2 cells were quantified (right panel). (d) ELISA assay for IL‐6 expression in tumour lysates. MPs: macrophages; NPs, neutrophils. All quantitative data was presented as mean ± standard deviation, *p < .05; **p < .01 vs. WT + PBS group. # p < .05; ## p < .01 vs. KO + PBS group; two‐tailed student t‐test. Figure S4. Lack of SIRPα reduced activation of SHP‐1, p38 MAPK and STAT3 in BMDMs and TAMs. (a) Quantitative analysis of Western blot results for the expression of SIRPα, p‐SHP‐1, p‐p38 MAPK and [file CPR-56-e13361-s002.docx]
