# Supplementary material for: Lack of SIRP‐alpha reduces lung cancer growth in mice by promoting anti‐tumour ability of macrophages and neutrophils
Source: Cell Prolif. 2022 Nov 23;56(2):e13361. doi: 10.1111/cpr.13361 (PMC9890530; doi:10.1111/cpr.13361)
Supplement: Supplementary file 2 — Table S1. Information of antibodies used for western blot (WB), immunofluorescence (IF) and enzyme‐linked immunosorbent assay (ELISA). Table S2. Primers used in manuscript. [file CPR-56-e13361-s001.docx]

**Supplemental Table 1. Information of antibodies used for western blot (WB), immunofluorescence (IF) and enzyme-linked immunosorbent assay (ELISA)**

| **Ab name** | **Company** | **identifier** | **Source** | **Dillution** | **Note** |
| --- | --- | --- | --- | --- | --- |
| Sirpα | Biolengend | Cat#144036 | Rat | 1:500 | WB |
| E-cadherin | Abcam | Cat#ab40772 | Rabbit | 1:8000 | WB |
| N-cadherin | Cell Signaling  Technology | Cat#13166 | Rabbit | 1:1000 | WB |
| Vimentin | Cell Signaling  Technology | Cat#5741 | Rabbit | 1:1000 | WB |
| Snail | Cell Signaling  Technology | Cat#3879 | Rabbit | 1:1000 | WB |
| F4/80 | Santa Cruz  Biotechnology | Cat#sc-377009 | Mouse | 1:50 | IF |
| p-STAT3  (Tyr705) | Abmart | Cat#T56566 | Rabbit | 1:5000  1:500 | WB  IF |
| STAT3 | Cell Signaling  Technology | Cat#4907 | Rabbit | 1:1000 | WB |
| ERK2 | Santa Cruz  Biotechnology | Cat#sc-154 | Rabbit | 1:3000 | WB |
| p-ERK1/2 | Cell Signaling  Technology | Cat#4370 | Rabbit | 1:2000 | WB |
| MAPK P38 | Absin | Cat#abs155950 | Rabbit | 1:1000 | WB |
| MAPK P-P38 | Santa Cruz  Biotechnology | Cat#11581 | Rabbit | 1:200  1:50 | WB  IF |
| p-SHP-1 | Absin | Cat#abs130871 | Rabbit | 1:1000  1:200 | WB  IF |
| SHP-1 | Abcam | Cat#ab32559 | Rabbit | 1:1000 | WB |
| IL-6 | R&D systems | Cat#MAB406 | Rat | 1:200  1:200 | IF  ELISA |
| TNF-α | R&D systems | Cat#MAB4102 | Rat | 1:200 | ELISA |
| GAPDH | proteintech | Cat#60004-1-Ig | Mouse | 1:8000 | WB |
| anti-mouse IgG  HRP-linked  antibody | Absin | Cat#abs20039 | Goat | 1:2000 | WB |
| anti-rabbit IgG  HRP-linked  antibody | Cell Signaling  Technology | Cat#7074 | Goat | 1:1000 | WB |
| anti-rat IgG  HRP-linked  antibody | Absin | Cat#abs20031 | Goat | 1:10000 | WB |
| Cy3-conjugated  Anti-Rabbit  IgG(H+L) | Jackson immunoResearch | Cat#711165152 | Donkey | 1:200 | IF |
| Cy3-conjugated  Anti-Mouse  IgG(H+L) | Jackson immunoResearch | Cat#5151165003 | Donkey | 1:200 | IF |
| FITC-conjugated  Anti-Mouse  IgG(H+L) | WELLBI | Cat#WH1115 | Goat | 1:200 | IF |
| PE Streptavidin | BD Bioscience | Cat#554061 | / | 1:200 | IF |

**Supplemental Table 2. Primers used in manuscript**

| Gene | Forward | Reverse |
| --- | --- | --- |
| β-actin | CCTTCTTGGGTATGGAATCCTGT | GGCATAGAGGTCTTTACGGATGT |
| Sirpα | CTCTCCGCGTCCTGTTTCTG | TCTGTACCACCTAATGGGTCC |
| IL-6 | CAACGATGATGCACTTGC | GTACTCCAGGTAGCTATG |
| Rantes | TCGTGCCCACGTCAAGGAGT | ACTAGAGCAAGCAATGACAG |
| Arginase-1 | CATTGGCTTGCGAGACGTAGAC | GCTGAAGGTCTCTTCCATCACC |
| TLR4 | GCCTTTCAGGGAATTAAGCTCC | GATCAACCGATGGACGTGTAAA |
| CCL2 | CACTCACCTGCTGCTACTCATTCAC | CTTCTTTGGGACACCTGCTGCTG |
| CXCL1 | GGCTGGGATTCACCTCAAGAACATC | TGAGTGTGGCTATGACTTCGGTTTG |
| PD-1 | GGTATCCCTGTATTGCTGCTGCTG | CTTCAGAGTGTCGTCCTTGCTTCC |
| PD-L1 | CTGGACCTGCTTGCGTTAGTGG | CCCCTGAAGTTGCTGTGCTGAG |
| Granzyme B | GTGCTGACTGCTGCTCACTGTG | TTGCTGGGTCTTCTCCTGTTCTTTG |
| Perforin | CTCCTCCTATGGCACGCACTTTATC | TTCAGGCAGTCTCCTACCTCATCAG |
| IFN-γ | CTGGAGGAACTGGCAAAAGGATGG | GACGCTTATGTTGTTGCTGATGGC |
